# Transient characteristics of universal cells on human‐induced pluripotent stem cells and their differentiated cells derived from foetal stem cells with mixed donor sources

**DOI:** 10.1111/cpr.12995

**Published:** 2021-02-01

**Authors:** Tzu‐Cheng Sung, Yi‐Peng Jiang, Jhe‐Yu Hsu, Qing‐Dong Ling, Hao Chen, Suresh S. Kumar, Yung Chang, Shih‐Tien Hsu, Qingsong Ye, Akon Higuchi

**Affiliations:** ^1^ School of Ophthalmology and Optometry Eye Hospital Wenzhou Medical University Wenzhou China; ^2^ Department of Chemical and Materials Engineering National Central University Taoyuan Taiwan; ^3^ Cathay Medical Research Institute Cathay General Hospital Taipei Taiwan; ^4^ Department of Biotechnology Bharath Institute of Higher Education and Research Chennai India; ^5^ Department of Chemical Engineering and R&D Center for Membrane Technology Chung Yuan Christian University Taoyuan Taiwan; ^6^ Department of Internal Medicine Taiwan Landseed Hospital Pingjen City Taiwan; ^7^ Center of Regenerative Medicine Renmin Hospital of Wuhan University Hubei China; ^8^ School and Hospital of Stomatology Wenzhou Medical University Wenzhou China; ^9^ Department of Oral Maxillofacial Surgery Skeletal Biology Research Center Massachusetts General Hospital Harvard School of Dental Medicine Boston MA USA; ^10^ Wenzhou Institute University of Chinese Academy of Science Wenzhou China; ^11^ Nano Medical Engineering Laboratory Riken Cluster for Pioneering Research Riken Japan

**Keywords:** human‐induced pluripotent stem cells, regenerative medicine, stem cells, universal cells

## Abstract

**Introduction:**

It is important to prepare ‘hypoimmunogenic’ or ‘universal’ human pluripotent stem cells (hPSCs) with gene‐editing technology by knocking out or in immune‐related genes, because only a few hypoimmunogenic or universal hPSC lines would be sufficient to store for their off‐the‐shelf use. However, these hypoimmunogenic or universal hPSCs prepared previously were all genetically edited, which makes laborious processes to check and evaluate no abnormal gene editing of hPSCs.

**Methods:**

Universal human‐induced pluripotent stem cells (hiPSCs) were generated without gene editing, which were reprogrammed from foetal stem cells (human amniotic fluid stem cells) with mixing 2‐5 allogenic donors but not with single donor. We evaluated human leucocyte antigen (HLA)‐expressing class Ia and class II of our hiPSCs and their differentiated cells into embryoid bodies, cardiomyocytes and mesenchymal stem cells. We further evaluated immunogenic response of transient universal hiPSCs with allogenic mononuclear cells from survival rate and cytokine production, which were generated by the cells due to immunogenic reactions.

**Results:**

Our universal hiPSCs during passages 10‐25 did not have immunogenic reaction from allogenic mononuclear cells even after differentiation into cardiomyocytes, embryoid bodies and mesenchymal stem cells. Furthermore, the cells including the differentiated cells did not express HLA class Ia and class II. Cardiomyocytes differentiated from transient universal hiPSCs at passage 21‐22 survived and continued beating even after treatment with allogenic mononuclear cells.

## Graphical abstract

Universal (hypoimmunogenic) human‐induced pluripotent stem cells (hiPSCs) were generated without gene editing, which were reprogrammed from foetal stem cells (human amniotic fluid stem cells) with mixing 2‐5 allogenic donors but not with single donor. Our universal hiPSCs during passages 10‐25 did not have immunogenic reaction from allogenic mononuclear cells including CD8^+^ killer T cells, natural killer (NK) cells, CD4^+^helper T cells and macrophages even after differentiation into cardiomyocytes, embryoid bodies and mesenchymal stem cells, whereas significant numbers of dead cells were observed for human embryonic stem cells (H9), human‐induced pluripotent stem cells (hiPSCs, HPS0077) and hiPSCs prepared from amniotic fluid stem cells with single donor as well as their differentiated cells.

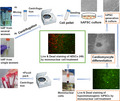

## 1 INTRODUCTION

Millions of people suffer from loss and injury of tissue and organs each year because of accidents, diseases and birth defects. Stem cells are an attractive source of cells for cell therapy.^1^, ^2^, ^3^, ^4^ Pluripotent stem cells (PSCs), such as embryonic stem cells (ESCs), have the potential to differentiate into any of the cell types derived from the 3 germ layers.^5^, ^6^, ^7^ However, the disadvantage of human ESC (hESCs) therapy is that large numbers of different types of hESCs with specific human leucocyte antigen (HLA) class I and class II types should be prepared and banked for therapy. This is because the hESCs used for transplantation should match the HLA type of the recipient, which generates an extremely high cost for hESC therapy; this will lead to a high cost in the National Insurance Budget once hESC therapy is covered by National Insurance in each country.

Recently, human PSCs (hPSCs) similar to hESCs were obtained from adult somatic cells by inducing ‘forced’ expression of certain pluripotency‐related genes,^8^ such as *Sox2*, *Oct3/4*, *klf‐4*, and *c‐myc* or *l‐myc*, miRNAs^9^ or proteins (protein‐induced PSCs),^10^, ^11^ which can be generated from patient cells for treatment.^11^ Such cells are known as human‐induced pluripotent stem cells (hiPSCs).

However, it takes time to generate mature hiPSCs, and hiPSCs require testing to verify that there is no gene abnormality and no contamination with viruses or other pathogens, which leads to a high cost of hiPSC therapy, with laborious and time‐consuming processes to generate patient‐specific hiPSCs from somatic cells from each patient. However, the hiPSCs derived from each patient do not generate immunogenicity‐related problems in general after the transplantation of differentiated cells from these hiPSCs.

To reduce the high cost of preparation for hESCs and hiPSCs that match the HLA types of specific patients, the banking of hPSC lines, which are clinically approved, is proposed to provide a source of hPSCs. Although it has been reported that hESCs show relatively low expression levels of major histocompatibility complex (MHC, HLA class I and class II) proteins compared to those of normal somatic cells,^12^, ^13^, ^14^ Taylor and colleagues suggested that the development of approximately 150 homozygous hESC lines would be necessary to provide sufficient HLA types to match hESC derivatives for most patients in the UK.^15^, ^16^ Nakajima and his colleagues also suggested that 170 homozygous hESC lines would support HLA matching for 80% of patients in Japan (but not 100% of patients).^17^ It is necessary to develop hPSCs that do not or less express HLA class Ia (HLA‐A, ‐B and ‐C) and class II (universal or hypoimmunogenic hPSCs) even after differentiation into specific lineages of cells, as a single or few hPSC cell lines could theoretically be used to treat every patient using stem cell therapy. In this case, universal or hypoimmunogenic hPSCs should not or less express HLA class Ia and class II even after they are differentiated into several specific lineages, which are used for stem cell therapy. However, it should be noted that hPSCs expressing no HLA class I such as the cells knocked out β2‐microglobulin (an important component of HLA class I) leads to the missing‐self response, which activate the lysing of the cells by natural killer (NK) cells.^15^, ^18^, ^19^, ^20^, ^21^, ^22^, ^23^ Therefore, several researchers developed (a) hPSCs where HLA‐A, ‐B and/or ‐C expression were edited to be homozygous or knocked out,^18^, ^20^, ^23^, ^24^, ^25^ (b) hPSCs where HLA class Ia (HLA‐A, HLA‐B and HLA‐C) was knocked out and HLA‐E, HLA‐G, PD‐L1 and/or CD47 genes were knocked in^19^, ^21^ or (c) hPSCs, which express HLA‐G, PD‐L1, CTLA4‐Ig and/or CD47 genes extensively^26^, ^27^ for universal or hypoimmunogenic hPSCs to be covered for any patient treatment with few cell lines of hPSCs. These universal or hypoimmunogenic hPSCs developed previously were all genetically modified, which makes laborious processes to evaluate and check no abnormal gene editing of hPSCs.

Here, we report generation method of universal hiPSCs from multiple sources of human amniotic fluids without gene editing, which have immune privilege from allogenic mononuclear cells containing CD8^+^ killer T cells, NK cells, CD4^+^ helper T cells and macrophages during passage 10‐25 even after differentiation into cardiomyocytes, embryoid bodies and mesenchymal stem cells. Our universal hiPSCs during passage 10‐25 and their differentiated cells do not express HLA class Ia and class II and may theoretically be used to treat any patient using only 1 cell line in clinical application in future.

## 2 MATERIALS AND METHODS

### 2.1 Ethical statement

All experiments in this study were approved by the ethics committees of Cathay General Hospital (CGH‐P108082), Taiwan Landseed Hospital (LSIHIRB 18‐009‐A2) and National Central University. All animal procedures were performed in strict accordance to the Guide for the Care and Use of Laboratory Animals and approved by the Institutional Animal Care and Use Committee (National Central University, Jhongli, Taiwan). All experiments were performed in accordance with all relevant and applicable governmental and institutional guidelines and regulations.

### 2.2 Materials

Human ESCs (H9, WiCell Research Institute, Inc) and hiPSCs (HPS0077, Riken BioResource Center) were used as control cell sources in this research. The materials used in this project are shown in Table S1. The other chemicals that were used in this research were obtained from Sigma‐Aldrich.

### 2.3 Isolation and culture of human amniotic fluid stem cells from amniotic fluid

**FIGURE 1:**
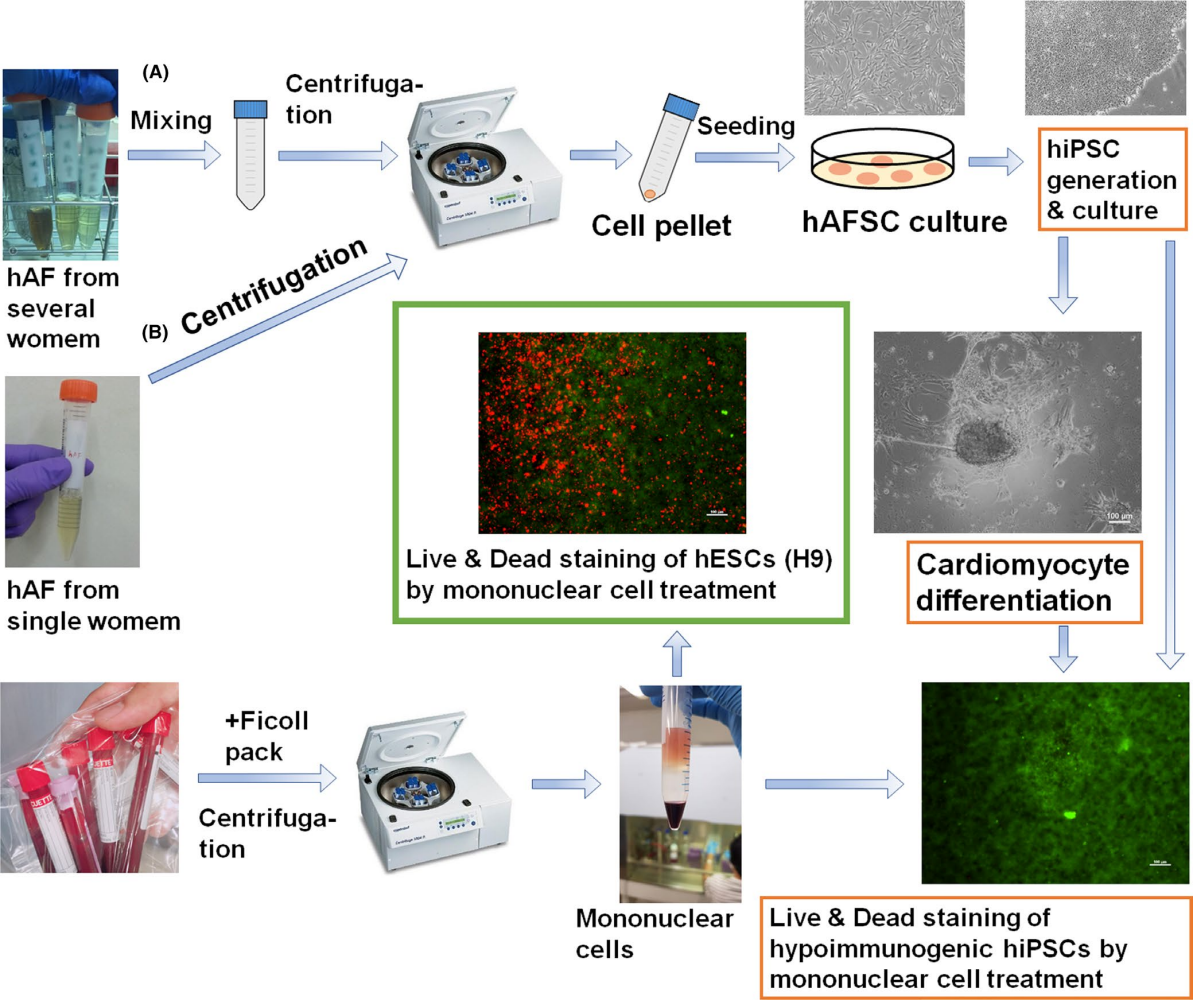
Outline of the process of reprogramming hAFSCs into transient universal (hypoimmunogenic) hiPSCs prepared from multiple AF donors (A) and conventional hiPSCs prepared from a single AF donor (B). The immunotolerance of transient universal (hypoimmunogenic) hiPSCs and their differentiated cardiomyocytes or embryoid bodies (EBs) is evaluated after treatment with mononuclear cells donated by different AF donors

Second‐trimester amniotic fluid (AF) derived from a single donor (a) or second‐trimester AF derived from multiple donors (2 or 5 pregnant women), which were mixed and stored for more than 2 days in 25°C (b), were used. The AF was centrifuged at 255 × *g* for 300 seconds, and the supernatant was discarded (Figure 1). The AF cells (cell pellets) were suspended in medium composed of MCDB 201/DMEM (60%/40%) supplemented with 10 ng/mL FGF‐2 and 20% foetal bovine serum (FBS) and cultivated on tissue culture polystyrene (TCPS) plates in a CO_2_ incubator at 37°C to obtain human amniotic fluid stem cells (hAFSCs).^28^ After reaching approximately 78%‐82% confluence, the cells (hAFSCs) were detached with a 0.25% trypsin‐EDTA solution, centrifuged and inoculated into TCPS dishes according to a conventional passage procedure (Figure 1).^28^ hAFSCs at passage 3‐4 were used for the following reprogramming processes.

### 2.4 Isolation and culture of human adipose‐derived stem cells from human fat tissue

Human adipose‐derived stem cells (hADSCs) were isolated from the fat pads from the omentum of 4 human patients (46‐67 years old) after each patient provided his or her informed consent in writing. The adipose tissue cell solution (stromal vascular fraction; SVF) was obtained following a conventional method.^29^, ^30^ The cells in the adipose tissue cell solution were seeded in medium composed of DMEM supplemented with 10% FBS to obtain hADSCs and cultured for 4 passages.^29^, ^30^ The medium was changed every other day.

### 2.5 Reprogramming of hAFSCs into hiPSCs

The CytoTune^R^‐iPS 2.0 Sendai Reprogramming Kit that included 3 SeV‐based reprogramming vectors was utilized for safe reprogramming from hAFSCs, which were obtained by mixing AF from 2‐5 donors or a single donor, into hiPSCs in this study. The protocol for hiPSC generation by reprogramming of hAFSCs using Sendai virus vector followed the manufacturer's protocol.^31^

### 2.6 Generation of transient universal hESCs

Timeline for the generation method of transient universal hESCs is shown in Figure S7A. After obtaining informed consent, mononuclear cells were isolated from blood from volunteers using Ficoll‐Paque solution and a conventional method.^32^, ^33^ On day −7, hESCs (H9) were inoculated into Matrigel‐coated plates and cultured in Essential 8 (E8) medium until day 0 by exchanging the E8 medium every day. On day 0, hESCs were detached from Matrigel‐coated dishes using dispase from a conventional protocol. Mononuclear cells (1.0 × 10^5^ cells) were inserted into centrifugation tubes together with hESCs (1.0 × 10^5^ cells) in 1 mL E8 medium and were incubated at 25°C for 2 days. On day 2, the cells were inoculated on Matrigel‐coated dishes and cultivated in E8 medium for 7 days by exchanging the E8 medium every day. On day 9, hESCs were detached from Matrigel‐coated dishes using dispase from a conventional protocol. Mononuclear cells (1.0 × 10^5^ cells) were inserted into centrifugation tubes together with hESCs (1.0 × 10^5^ cells) in 1 mL E8 medium and were incubated at 25°C for 2 days. On day 11, the cells were inoculated on Matrigel‐coated dishes and cultivated in E8 medium for 7 days by exchanging the E8 medium every day. On day 18, hESCs were detached from Matrigel‐coated dishes using dispase from a conventional protocol. Mononuclear cells (1.0 × 10^5^ cells) were inserted into centrifugation tubes together with hESCs (1.0 × 10^5^ cells) in 1 mL E8 medium and were incubated at 25°C for 2 days. On day 20, the cells were inoculated on Matrigel‐coated dishes and cultivated in E8 medium by exchanging the E8 medium every day for several passages. After day 27, the cells are regarded as transient universal hESCs.

### 2.7 Culture and characterization of hESCs and hiPSCs

hESCs (H9), hiPSCs (HPS0077), hiPSCs (single) and universal hiPSCs were maintained on recombinant vitronectin‐coated TCPS dishes in Essential 8 medium.^31^, ^34^, ^35^, ^36^ The medium was exchanged daily during the experiments.

Immunostaining for Sox2, Nanog, SSEA‐4, and Oct3/4 was performed on hPSCs to investigate pluripotency according to a conventional protocol.^31^, ^34^, ^35^, ^36^ The stained cells were evaluated using fluorescence microscopy.

hPSC pluripotency was studied by embryoid bodies (EBs) formation. hPSCs were dissociated from the cell culture plates. hPSCs in the supernatant were collected, counted utilizing a hemocytometer, and plated onto ultralow attachment plates in Essential 6 medium to form EBs. After 2 weeks in suspension, the EBs were shifted into Matrigel‐coated plates and cultivated for an additional 4 weeks. Subsequently, the cells were stained with antibodies against markers of all 3 embryonic germline layers (glial fibrillary acidic protein (GFAP), α‐fetoprotein (AFP), and smooth muscle actin (SMA)) utilizing a standard protocol and evaluated with an immunofluorescence method.^31^, ^34^, ^35^, ^36^

hPSCs were collected by treating the cells with a nonenzymatic cell dissociation solution. The cell pellets were suspended in Dulbecco's modified Eagle's medium (DMEM/F12) medium with Matrigel after centrifuging the cells. A total of 5.0 × 10^6^ cells were injected subcutaneously into NOD.CB17‐Prkd^cscid^/JNarl NOD/SCID (nonobese diabetic/severe combined immunodeficiency) mice. After 8 weeks, the developed teratomas were dissected and fixed in formaldehyde solution. Paraffin‐embedded teratomas were sectioned and stained with haematoxylin and eosin (H&E) solution utilizing a standard protocol.^31^, ^34^, ^35^, ^36^

### 2.8 hPSC differentiation into cardiomyocytes

The hPSCs were differentiated into cardiomyocytes using a method developed by Sharma and colleagues^37^ with some modifications. On day −4, hPSCs were inoculated into Matrigel‐coated plates and cultured in Essential 8 medium until day 0 by exchanging the Essential 8 medium every day. On day 0, the hPSCs were approximately 80%‐85% confluent. The Essential 8 medium was changed to Roswell Park Memorial Institute (RPMI)‐1640 medium supplemented with 2 wt% B27 minus insulin and 6 μM CHIR99021 (a GSK‐3β inhibitor). The hPSCs were incubated for 2 days. On day 2, the medium was changed to RPMI‐1640 medium supplemented with 2 wt% B27 minus insulin, and the cells were incubated for 2 days. On day 4, the medium was changed to RPMI‐1640 medium supplemented with 5 μM IWR‐1 (a Wnt inhibitor) and 2 wt% B27 minus insulin, and the hPSCs were incubated for 2 days. On day 6, the medium was changed to RPMI‐1640 medium supplemented with 2 wt% B27 minus insulin, and the hPSCs were cultured for 1 day. On day 7, the medium was changed to RPMI‐1640 medium supplemented with 2 wt% B27. The hPSCs were cultured until day 18, with the medium replaced every other day.

### 2.9 hPSC differentiation into mesenchymal stem cells

The hPSCs were differentiated into mesenchymal stem cells (MSCs) using a method developed by Li and colleagues^38^ with some modifications. On day −4, hPSCs were inoculated into Matrigel‐coated plates and cultured in Essential 8 medium until day 1 by exchanging the Essential 8 medium every day. On day 1, the hPSCs were approximately 80%‐90% confluent. The Essential 8 medium was exchanged to Essential 6 medium supplemented with 10 ng/mL BMP4 and 1 μM/L A83‐01. At day 3, the cells were washed with phosphate‐buffered saline (PBS) and the medium was exchanged with MSC medium (αMEM containing 20% FBS). The cells were cultured in MSC medium for 10 passages. The medium was exchanged every other day.

### 2.10 HLA class Ia and class II expression of cells

The HLA Class Ia and Class II expression of hAFSCs, colon cancer cell lines (LoVo, Colo205, SW480 and HT29), hADSCs, hESCs (H9), hiPSCs (HPS0077), hiPSCs (single) and universal hiPSCs (mix) was evaluated using flow cytometry. The cells were incubated with anti‐HLA class Ia (1:500 dilution), anti‐HLA class II (1:500 dilution) or isotype antibodies (1:500 dilution) for 45 minutes, and the solution was centrifuged at 450 × *g* for 8 minutes. The cells were shifted into phosphate‐buffered saline solution and evaluated utilizing flow cytometry (BD Accuri™ C6; BD Biosciences).

### 2.11 Survival and cytokine production of hPSCs and hPSC‐derived cardiomyocytes after treatment with mononuclear cells

After obtaining informed consent, mononuclear cells were isolated from blood from male volunteers using Ficoll‐Paque solution and a conventional method.^32^, ^33^ Mononuclear cells were inserted into each cell culture plate of hAFSCs, hESCs (H9), HPS0077 (hiPSCs) and universal hiPSCs (mix) (10^4^ mononuclear cells/cm^2^ on TCPS dishes with a 3.5 cm diameter), and the cells were cultivated for 2 days. Cell survival was analysed with a live/dead staining kit following the manufacturer's recommended protocol. Live (green colour) and dead (red colour) cells were evaluated by fluorescence microscopy (Eclipse Ti‐U inverted fluorescence microscope; Nikon Instruments, Inc). The cell survival rate of the cells was also analysed by flow cytometry after the cells were stained with 7‐AAD.^32^, ^33^

### 2.12 Statistical analysis

All of the quantitative results were obtained from 4 samples. The data were presented as the means ± standard deviations (SDs). Statistical analysis was performed utilizing unpaired Student's *t* tests in Excel (Microsoft Corporation). Probability values (*P*) less than .05 were regarded as statistically significant.

## 3 RESULTS

### 3.1 HLA class Ia and class II expression of several human stem cells and cancer cells

**FIGURE 2:**
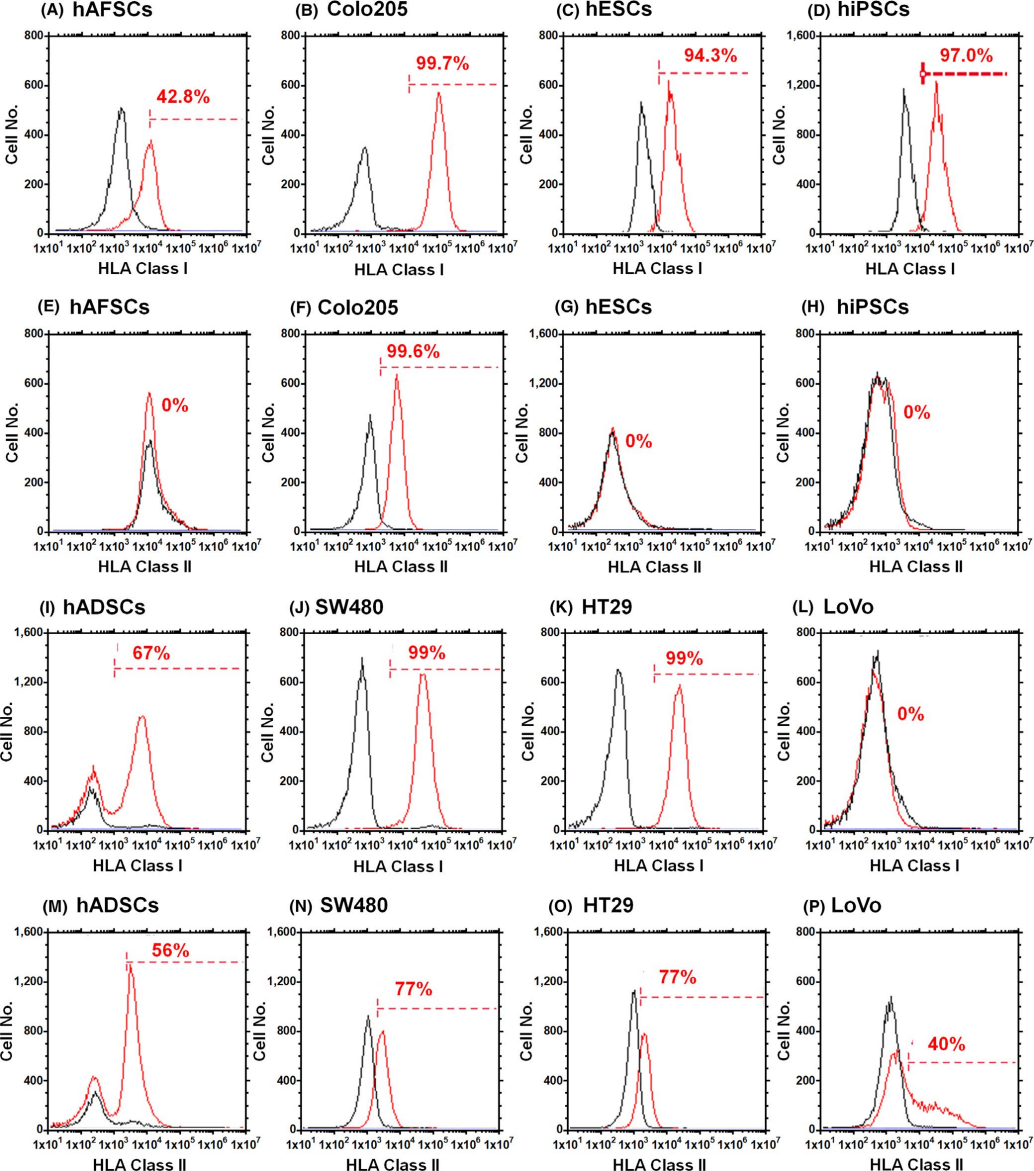
Expression of HLA class Ia and II on several human stem cells and cancer cells. Expression of HLA class Ia (A‐D, I‐L) and class II (E‐H, M‐P) on hAFSCs (A, E), Colo205 cells (B, F), hESCs (H9) (C, G), hiPSCs (HPS0077) (D, H), hADSCs (I, M), SW480 cells (J, N), HT29 cells (K, O) and LoVo cells (L, P) evaluated by flow cytometry. The black line indicates isotype staining of the cells

To prepare universal or hypoimmunogenic hiPSCs, we hypothesized that hiPSCs derived from some cell lines, such as foetal cells, for example, hAFSCs, might show some characteristics of universal hiPSCs. Therefore, the HLA class Ia and class II expression of several cell lines that we have stocked in our laboratory, such as hADSCs, LoVo cells, HT29 cells, SW480 cells, CoLo205, hESCs (H9), hiPSCs derived from fibroblasts (HPS0077) and hAFSCs, was investigated using flow cytometry to evaluate the potential use of these cells as mother cells to generate hiPSCs (Figure 2). hESCs and hiPSCs showed no HLA class II expression but expressed HLA class Ia, as reported by several other researchers.^12^, ^13^, ^14^ Furthermore, the differentiated cells from hESCs and hiPSCs expressed distinct HLA class Ia. Human ADSCs and most colon cancer cells, except LoVo cells, showed high expression of HLA class Ia. Because LoVo cells are a cancer cell line, we chose to use foetal stem cells of hAFSCs to generate hiPSCs using the Sendai virus vector.

### 3.2 Generation of transient universal hiPSCs

We generated transient universal hiPSCs from hAFSCs derived from multiple sources of AF, with AF from several pregnant women (2‐5 women) mixed to generate hAFSCs [hAFSCs (mix)] (Figure 1). We hypothesized that not all of the cells among the foetal stem cells express HLA class Ia and class II after reprogramming into hiPSCs. Furthermore, we expected that hAFSCs, which do not express HLA class Ia and class II after reprogramming, can be selected by treatment with AF derived from other donors and/or mononuclear cells that are contained in the AF derived from other donors.

**FIGURE 3:**
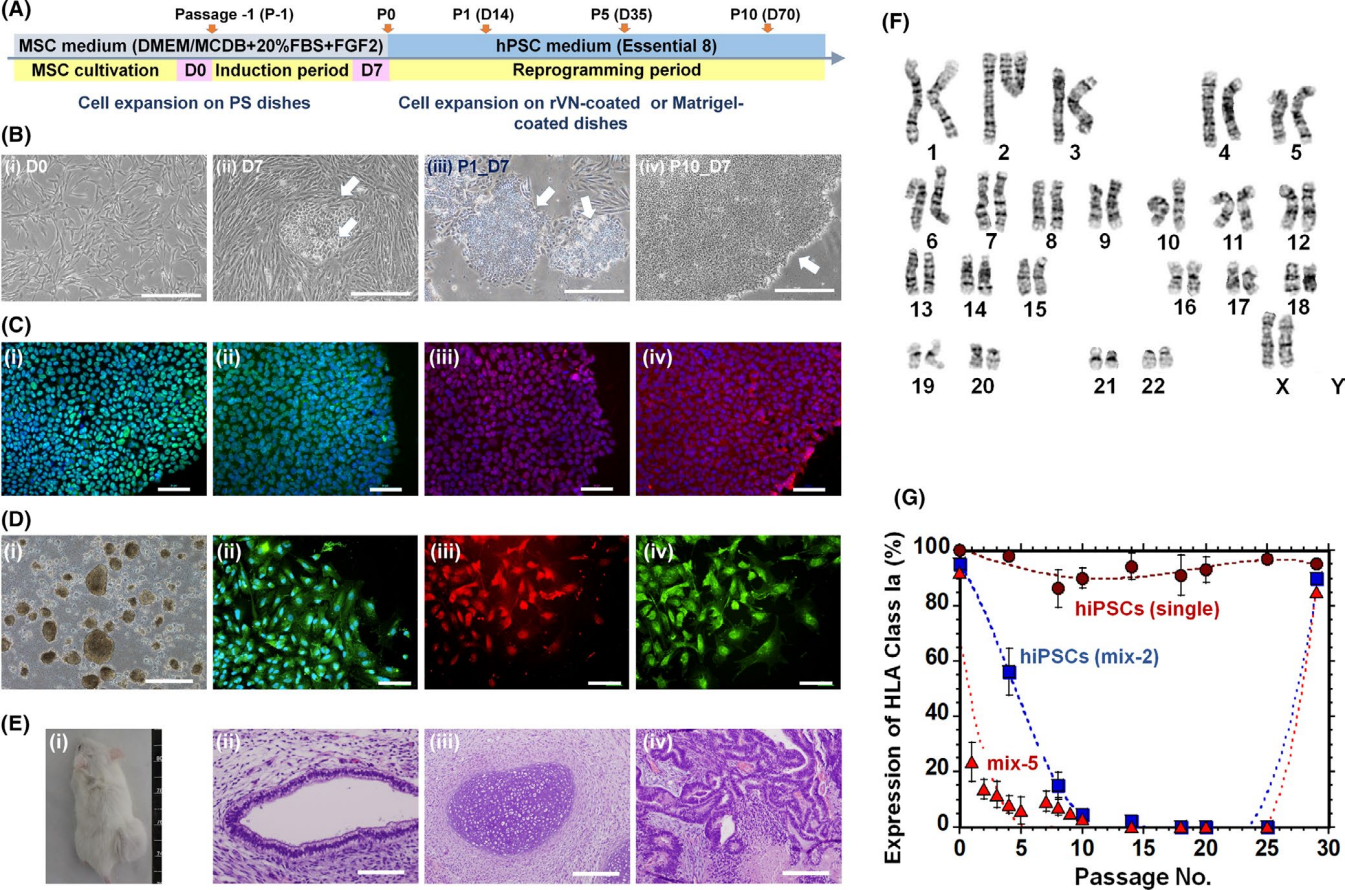
Generation of transient universal hiPSCs (mix). A, Preparation timeline of transient universal hiPSCs (mix) using hAFSCs. B, The sequential morphological changes during reprogramming of hAFSCs into transient universal hiPSCs (mix‐5) at day 0 of passage 0 (i), day 7 of passage 0 (ii), day 7 of passage 1 (iii) and day 7 of passage 10 (iv). The scale bar indicates 500 μm. C, Expression of the pluripotency proteins Oct4 (i, green), Sox2 (ii, green), Nanog (iii, red) and SSEA‐4 (iv, red) in transient universal hiPSCs (mix‐5) evaluated by immunostaining, with nuclear staining with Hoechst 33342 (blue) after culturing for 20 passages. The scale bar indicates 50 μm. D, (i) Morphology of cells from EBs differentiated from transient universal hiPSCs (mix‐5) after culturing for 21 passages. Expression of a mesodermal protein (ii, SMA, green), an ectodermal protein (iii, GFAP, red) and an endodermal protein (iv, AFP, green) in the cells shown by immunostaining, with nuclear staining with Hoechst 33342 (ii, blue), after culturing for 21 passages. The scale bar indicates 500 μm (i) and 100 μm (ii‐iv). E, A teratoma was formed by injecting transient universal hiPSCs (mix‐5) cultured on recombinant vitronectin‐coated dishes after 22 passages (i). Tissues including the gland duct consisting of the columnar epithelium (ii, endoderm), cartilage (iii, mesoderm) and immature neuroepithelium (iv, ectoderm) can be observed. The scale bar indicates 100 μm (ii) and 200 μm (iii, iv). F, Karyotyping of universal hiPSC (mix‐5) after culturing for 21 passages. G, Dependence of HLA class Ia expression of hiPSCs (single) (closed circle), hiPSCs (mix‐2) (closed square) and hiPSCs (mix‐5) (closed triangle) after reprogramming from hAFSCs on passage of the cells

We succeeded in generating hiPSCs using (a) hAFSCs derived from mixed AF [hiPSCs (mix)] and (b) hAFSCs from AF from a single donor [hiPSCs (single)] (Figures 3 and S1‐S4). We generated 2 types of transient universal hiPSCs, which were prepared using 2 different AF donors (hiPSCs (mix‐2)) and 5 different AF donors (hiPSCs (mix‐5)); these hiPSCs were generated from the transfection of pluripotency‐related genes (Yamanaka factors) using the Sendai virus vector. The hiPSC colonies were typically observed 7‐10 days after transfection of pluripotency‐related genes (Figures 3(B), S1B and S2B). The hiPSCs generated from hAFSCs derived from mixed AF and from single AF expressed pluripotency‐related proteins such as Oct3/4, Sox2, Nanog and SSEA‐4 (Figures 3(C), S1C, S2C and S3), as indicated by immunostaining evaluation of pluripotency‐related proteins. The hiPSCs had the ability to differentiate into cells derived from 3 germ layers in vitro (Figures 3(D), S1D, S2D and S4) and in vivo (Figures 3(E), S1E, and S2E), which are the main characteristics of hPSCs. We also evaluated karyotyping of the transient universal hiPSCs (mix‐5), and the results are shown in Figure 3(F). The karyotypes of the cells were found to be normal, suggesting that there were no genetic abnormalities on the cells during their reprogramming into universal hiPSCs (mix‐5). Surprisingly, the reprogrammed cells from mixed hAFSCs into hiPSCs (mix) gradually decreased their expression of HLA class Ia did not express HLA class Ia during 10‐25 passages, whereas high expression of HLA class Ia was observed for hiPSCs (single) at any passages (Figure 3G), although hAFSCs showed distinct expression of HLA class Ia (Figure 2A) before induction into universal hiPSCs (mix) [hAFSCs (mix)]. However, the universal hiPSCs (mix) start to express HLA class Ia after 29 passages. Therefore, current hiPSCs prepared with mixing of AFs from multi donors have transient characteristics of universal cells (no expression of HLA class Ia and II), which are limited to be the universal cells during 10‐25 passages. Furthermore, the key technology to generate transient universal hiPSCs is prepared from foetal stem cells derived from mixing of AFs from multi donors in this study, because the universal hiPSCs cannot generated from the reprogramming of hAFSCs derived from single donor (Figure 3G).

### 3.3 Differentiation of mixed AF‐derived hiPSCs into cardiomyocytes and their HLA class I and class II expression

**FIGURE 4:**
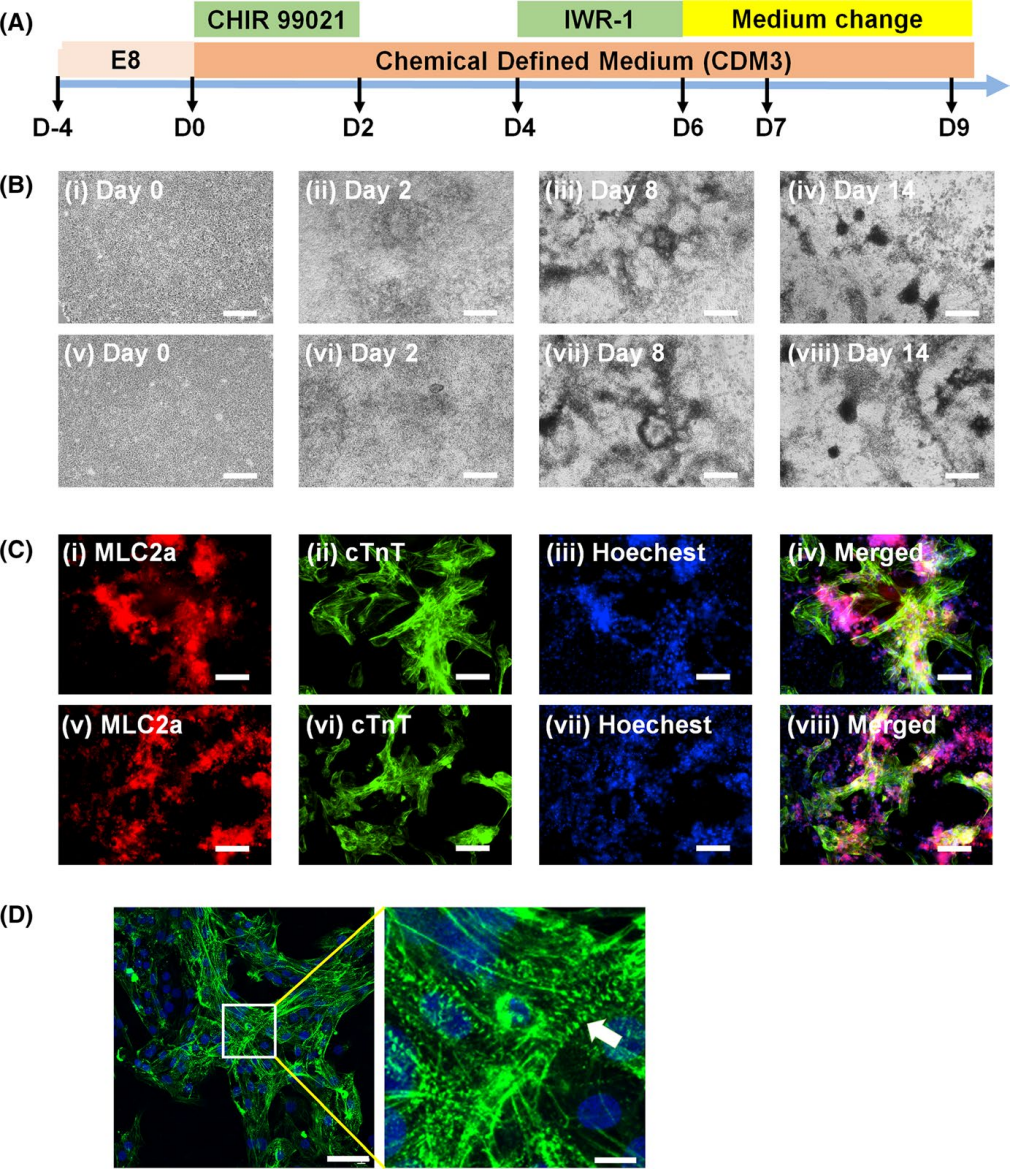
Cardiomyocyte differentiation of transient universal hiPSCs (mix‐5) at passage 22 and hiPSCs (mix‐2) at passage 21. A, Timeline of the protocol for hPSC differentiation into cardiomyocytes. B, Sequential morphological changes during the differentiation of universal hiPSCs (mix‐5) (i‐iv) and hiPSCs (mix‐2) (v‐viii) towards the cardiac lineage on days 0 (i, v), 2 (ii, vi), 8 (iii, vii) and 14 (iv, viii) after inducing the cells to differentiate into cardiomyocytes. The scale bar indicates 100 μm. C, (i, ii) The MLC2a (i) and cTnT (ii) expression levels were evaluated by fluorescence microscopy of differentiated cardiomyocytes derived from transient universal hiPSCs (mix‐5) on day 14. (iii) Hoechst 33342 staining of cardiomyocytes derived from transient universal hiPSCs (mix‐5) on day 14. Photograph (iv) was generated by merging (i)‐(iii). The MLC2a (v) and cTnT (vi) expression levels were evaluated by fluorescence microscopy of differentiated cardiomyocytes derived from transient universal hiPSCs (mix‐2) on day 14. (vii) Hoechst 33342 staining of cardiomyocytes derived from transient universal hiPSCs (mix‐2) on day 14. Photograph (viii) was generated by merging (v)‐(vii). The bar indicates 50 μm. D, (i) The α‐actinin expression levels were evaluated by confocal laser microscopy of differentiated transient universal hiPSCs (mix‐5)‐derived cardiomyocytes on day 14. The scale bars indicate 50 μm. (ii) A high‐magnification image of (i). The arrow indicates sarcomere units (with a ladder‐like morphology). The scale bars indicate 5 μm

The hiPSCs (mix) at passage 21‐22, which were derived from mixed AF from different women, and hiPSCs (single) at passage 20 from a single AF donor were induced to differentiate into cardiomyocytes (Figures 4 and S5). We used this approach because we wanted to evaluate the expression of HLA class Ia and class II after differentiation of our transient universal hiPSCs into a specific lineage of cells.

**FIGURE 5:**
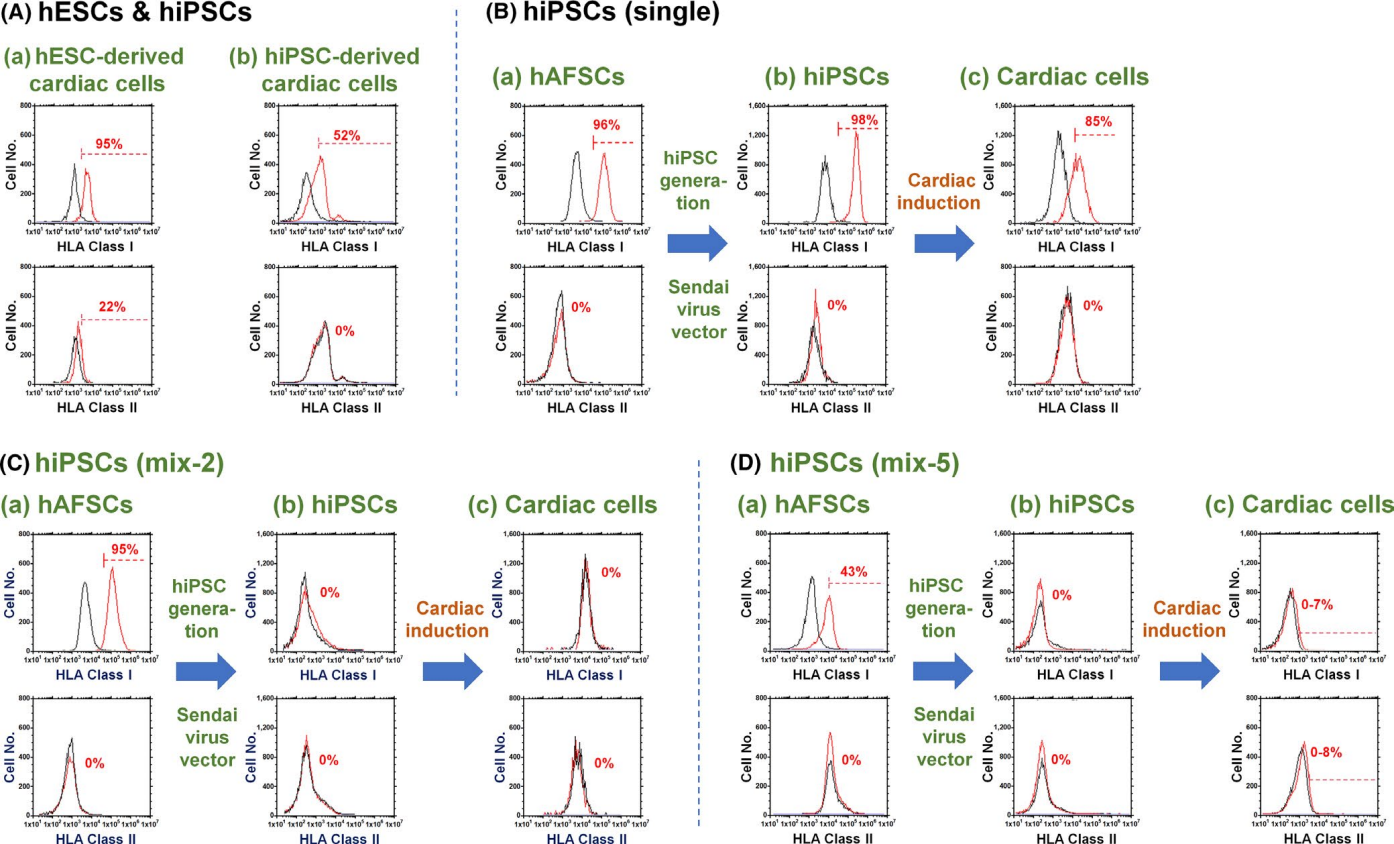
Expression of HLA class I and class II of hAFSCs, several hPSCs and their differentiated cells. A, HLA class Ia and class II expression of hESC (H9)‐derived cardiomyocytes (a) and hiPSC (HPS0077)‐derived cardiomyocytes (b) on day 14 of induction. B, HLA class Ia and class II expression of hAFSCs derived from a single AF donor (hAFSCs (single)) (a), hiPSCs (single) reprogrammed from hAFSCs (single) at passage 20 (b), and cardiomyocytes derived from hiPSCs (single) of passage 20 (c). C, HLA class Ia and class II expression of hAFSCs derived from mixing AF from 2 donors (hAFSCs (mix)) (a), universal hiPSCs (mix‐2) reprogrammed from hAFSCs (mix) at passage 21 (b), and cardiomyocytes derived from universal hiPSCs (mix‐2) of passage 21 (c). D, HLA class Ia and class II expression of hAFSCs derived from mixing AF from 5 donors (hAFSCs (mix)) (a), transient universal hiPSCs (mix‐5) reprogrammed from hAFSCs (mix) at passage 22 (b), and cardiomyocytes derived from transient universal hiPSCs (mix‐5) of passage 22 (c)

Cardiomyocytes derived from hiPSCs (mix) and hiPSCs (single) expressed the cardiac‐specific proteins MLC2a, cTnT and α‐actinin (Figures 4C,D and S5C). Beating cells were also obtained (Video S1 (cardiomyocytes derived from hiPSCs (mix‐2)) and Video S2 (cardiomyocytes derived from hiPSCs (mix‐5)). We evaluated the HLA class Ia and class II expression of hAFSCs (mix), hAFSCs (single), hiPSCs (mix), hiPSCs (single) and cardiomyocytes derived from hiPSCs (mix) and hiPSCs (single) as well as that of hESC (H9)‐derived cardiomyocytes and hiPSC (HPS0077)‐derived cardiomyocytes at day 20 of the differentiation and the results are shown in Figure 5. As we expected, cardiomyocyte‐derived hiPSCs (mix) at day 20 of the differentiation as well as undifferentiated hiPSCs (mix) at passage 20 did not show HLA class Ia and Class II expression (Figure 5C,D). On the other hand, hiPSCs (single) expressed distinct HLA class Ia, and cardiomyocytes derived from hiPSCs (single) also showed HLA class Ia expression (Figure 5B). We found that the mixing of AF from different donors to generate hAFSCs is a key technique to generate transient universal hiPSCs. In particular, we succeeded in generating transient universal hiPSCs by mixing AF from a minimum of 2 different pregnant women, as hiPSCs (mix‐2) and cardiomyocytes differentiated from hiPSCs (mix‐2) did not express HLA class Ia and class II (Figure 5C,D).

### 3.4 Differentiation of transient universal hiPSCs into MSCs and their HLA class Ia and class II expression

We further differentiated hiPSCs (mix‐5) at passage 25 into MSCs, which expressed MSC markers of CD44, CD73, CD90 and CD105 after differentiation into MSCs at passage 5 (Figure S6). MSCs derived from hiPSCs (mix‐5) at passage 25 also did not show HLA class Ia and class II.

### 3.5 No immunogenic response of transient universal hiPSCs with allogenic mononuclear cells

**FIGURE 6:**
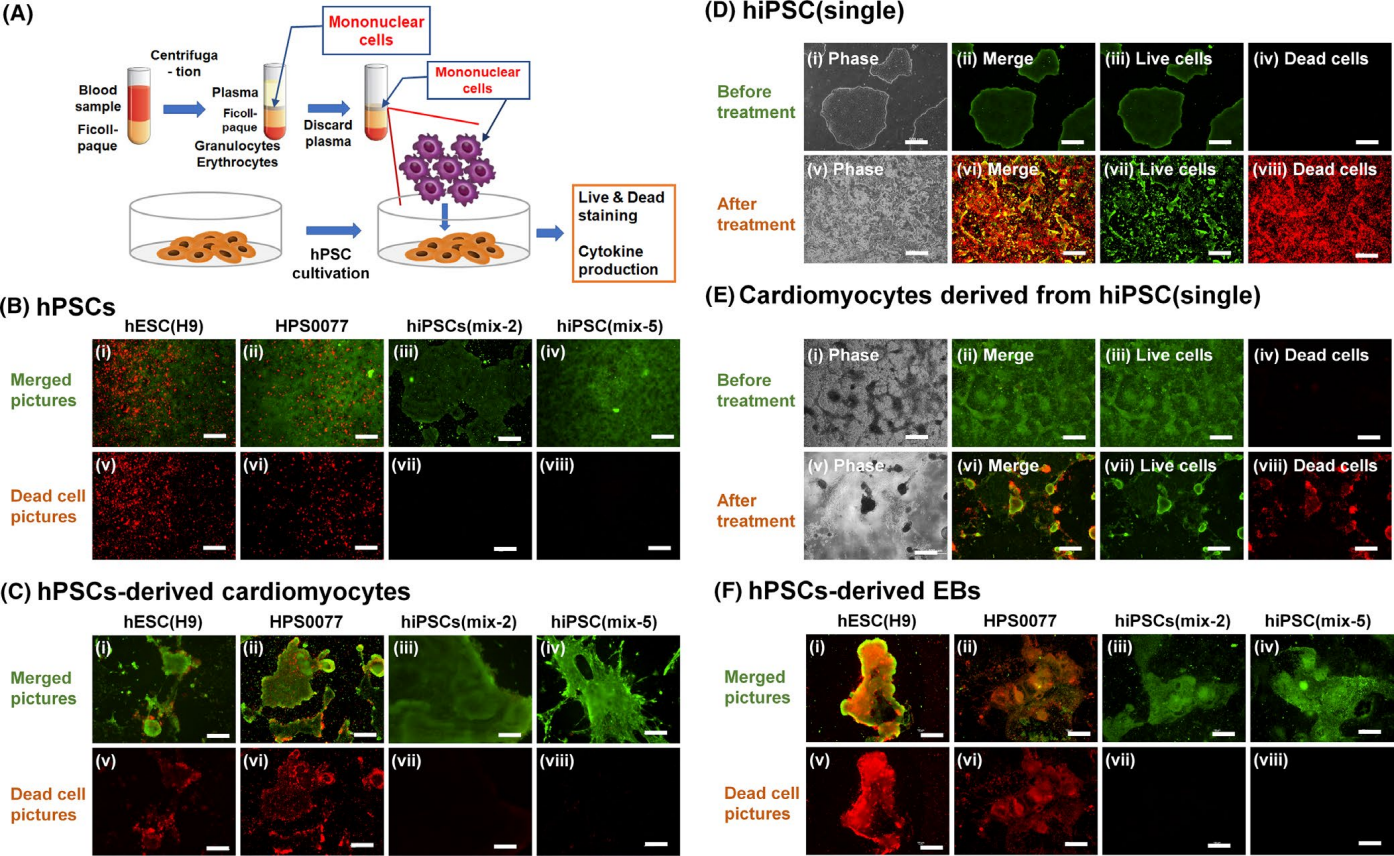
Evaluation of the immunogenic reaction of several hPSCs treated with mononuclear cells. A, Outline of immunogenic reaction experiments of hPSCs after contact with mononuclear cells. B, Live (green) and dead (red) staining of (i, v) hESCs (H9), (ii, vi) hiPSCs (HPS0077), (iii, vii) transient universal hiPSCs (mix‐2) at passage 20, which were reprogrammed from hAFSCs derived from multiple AF donors (2 donors), and (iv, viii) transient universal hiPSCs (mix‐5) at passage 20, which were reprogrammed from hAFSCs derived from multiple AF donors (5 donors), where the cells were treated with mononuclear cells for 2 days. The bar indicates 500 μm. C, Live (green) and dead (red) staining of cardiomyocytes derived from (i, v) hESCs (H9), (ii, vi) hiPSCs (HPS0077), (iii, vii) transient universal hiPSCs (mix‐2) at passage 21, and (iv, viii) transient universal hiPSCs (mix‐5) at passage 22, where the cells were treated with mononuclear cells for 2 days. The bar indicates 500 μm. D, E, Live (green, iii, vii) and dead (red, iv, viii) staining of (D) hiPSCs (single) and (E) cardiomyocytes derived from hiPSCs (single) at passage 20 before (i‐iv) and after (v‐viii) treatment of mononuclear cells for 2 days. (i, v) phase contrast images. Photograph (ii) was generated by merging (iii) and (iv). Photograph (vi) was generated by merging (vii) and (viii). The bar indicates 500 μm. F, Live (green) and dead (red) staining of EBs derived from (i, v) hESCs (H9), (ii, vi) hiPSCs (HPS0077), (iii, vii) transient universal hiPSCs (mix‐2) at passage 21, and (iv, viii) transient universal hiPSCs (mix‐5) at passage 21, where the cells were treated with mononuclear cells for 2 days. The bar indicates 500 μm

For future clinical application of transient universal hiPSCs, it is necessary to verify that our hiPSCs (mix) do not show immunogenic reactions with different sources of human cells (or tissue). For this purpose, we evaluated the cell viability of our hiPSCs (mix) treated with allogenic mononuclear cells, which were derived from different AF donors. The mononuclear cells derived from human peripheral blood are known to be consisted of (a) 40% CD3^+^CD4^+^ T cells, (b) 18% CD3^+^CD8^+^ T (activated T) cells, (c) 10% CD22^+^ B cells, (d) 15% NK cells, (e) 15% monocytes including macrophages approximately.^39^ The mononuclear cells (25 × 10^5^ cells) derived from human peripheral blood from volunteers were added to transient universal hiPSCs as well as cardiomyocytes differentiated from transient universal hiPSCs at passage 21‐22 and EBs (immature differentiated cells from 3 germ layers) derived from transient universal hiPSCs at passage 21 (5 × 10^5^ cells), and the cells were incubated for 2 days (Figure 6A). Cell viability was evaluated for hESCs (H9), hiPSCs (HPS0077), transient universal hiPSCs (mix‐2) at passage 20, transient universal hiPSCs (mix‐5) at passage 20 and hiPSCs (single) at passage 20 as well as cardiomyocytes and EBs derived from these hPSCs using live/dead staining (Figure 6B‐E). Human iPSCs (mix) (Figure 6B) and cardiomyocytes differentiated from transient universal hiPSCs (mix) (Figure 6C) showed high viability (green cells) and almost no dead cells (red cells), whereas significant numbers of dead cells were observed for undifferentiated hPSCs and cardiomyocytes differentiated from hESCs (H9), hiPSCs (HPS0077) and hiPSCs (single) (Figure 6B‐6E). EBs derived from hiPSCs (mix) also showed high viability after allogenic mononuclear cells were treated for 2 days (Figure 6F), whereas significant numbers of dead cells were observed for EBs derived from hESCs (H9) and hiPSCs (HPS0077) (Figure 6F). Therefore, the cells derived from 3 germ layers (endoderm, mesoderm and ectoderm) in EBs and cardiomyocytes, which were differentiated from hiPSCs (mix) as well as undifferentiated hiPSCs (mix), were found to have universal characteristics from these results during specific passages (10‐25 passages).

**FIGURE 7:**
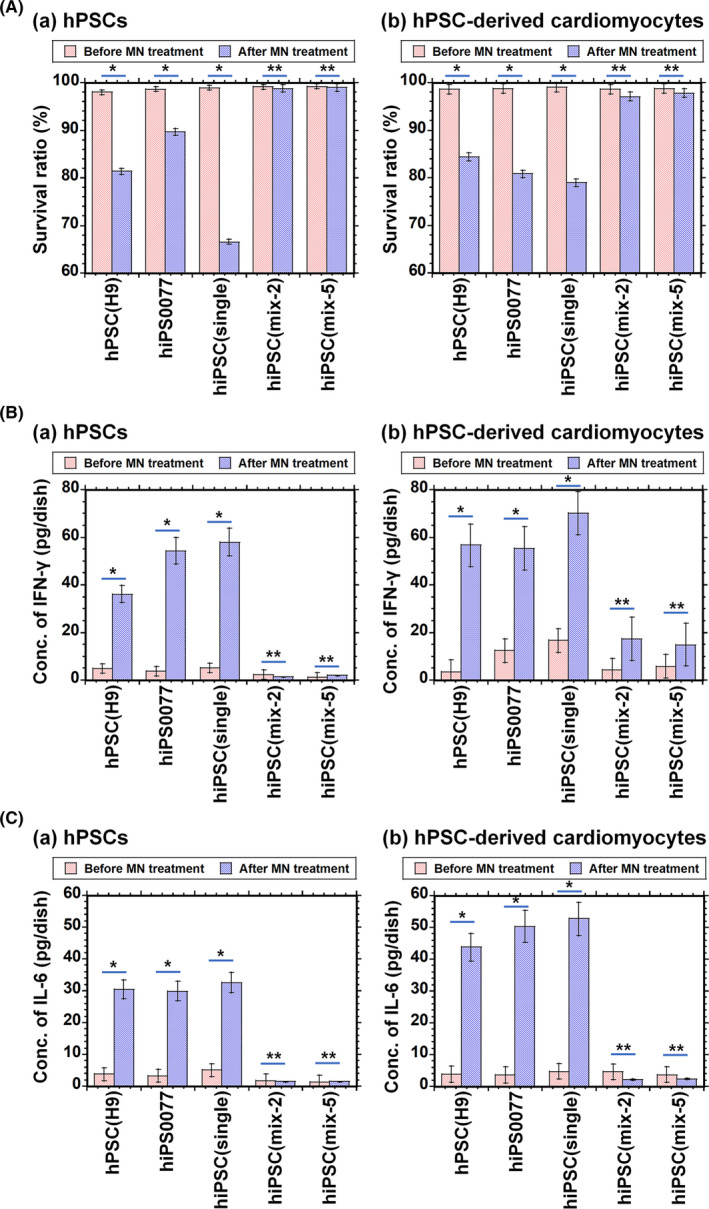
Immunogenic reaction of several hPSCs treated with mononuclear cells. A, (a) Survival rates of hESCs (H9), hiPSCs (HPS0077), hiPSCs (single) at passage 20, transient universal hiPSCs (mix‐2) at passage 20, and transient universal hiPSCs (mix‐5) at passage 20, which were calculated before (left red bar) and after (right blue bar) mononuclear cell treatment for 2 days at 37°C. (b) Survival rates of cardiomyocytes derived from hESCs (H9), hiPSCs (HPS0077), hiPSCs (single) at passage 20, transient universal hiPSCs (mix‐2) at passage 21 and transient universal hiPSCs (mix‐5) at passage 22, which were evaluated using flow cytometry before (left red bar) and after (right blue bar) mononuclear cell treatment for 2 days at 37°C. ^*^ *P* < .05, ^**^ *P* > .05. B, Production of interferon‐γ (IFN‐γ) by (a) hPSCs (hESCs (H9), hiPSCs (HPS0077), hiPSCs (single) at passage 20, transient universal hiPSCs (mix‐2) at passage 20 and transient universal hiPSCs (mix‐5) at passage 20) and (b) cardiomyocytes derived from these hPSCs before (left red bar) and after (right blue bar) mononuclear cell treatment for 2 days at 37°C. ^*^ *P* < .05. ^**^ *P* > .05. C, Production of interleukin‐6 (IL‐6) by (a) hPSCs (hESCs (H9), hiPSCs (HPS0077), hiPSCs (single) at passage 20, transient universal hiPSCs (mix‐2) at passage 20 and transient universal hiPSCs (mix‐5) at passage 20) and by (b) cardiomyocytes derived from these hPSCs before (left red bar) and after (right blue bar) mononuclear cell treatment for 2 days at 37°C. ^*^ *P* < .05, ^**^ *P* > .05

The survival rate under each condition was evaluated using flow cytometry after staining the cells with 7‐AAD, and the results are shown in Figure 7A. The survival rate was found to be almost 100% for transient universal hiPSCs (mix) at passage 20 and cardiomyocytes differentiated into transient universal hiPSCs (mix) at passage 21‐22 after treatment with mononuclear cells, whereas the survival rate of hiPSCs (HPS0077), hESCs (H9), hiPSCs (single) and cardiomyocytes differentiated from hiPSCs (HPS0077), hESCs (H9) or hiPSCs (single) were found to be much less than 90%. Human iPSCs (mix) and differentiated cells from hiPSCs (mix) at passage 20‐22 showed tolerance to mononuclear cells derived from different individuals.

Surprisingly, the cardiomyocytes differentiated from hiPSCs (mix) continued beating even after treatment with the mononuclear cells for more than 5 days (Videos S3 and S4 for cardiomyocytes derived from hiPSCs (mix‐2) and hiPSCs (mix‐5), respectively, after treatment with mononuclear cells), whereas the cardiomyocytes differentiated from conventional hESCs (H9), hiPSCs (HPS0077) and hiPSCs (single) stopped beating after treatment with the mononuclear cells.

We also evaluated cytokines generated by the cells due to immunogenic reactions between hESCs or hiPSCs and mononuclear cells derived from individuals different than those from whom the hESCs and hiPSCs originated. Figure 7B,C show interferon‐γ and interleukin 6 production, respectively, in the cell culture medium before and after the addition of mononuclear cells. Similar to the cell survival rate results, the culture medium containing (a) cardiomyocytes differentiated from hESCs and hiPSCs (HPS0077) and (b) hPSCs of conventional hESCs (H9) and hiPSCs (HPS0077) with mononuclear cells expressed high concentrations of these cytokines, whereas almost no cytokine production was observed in the culture medium containing cardiomyocytes differentiated from transient universal hiPSCs (mix) at passage 21‐22 or undifferentiated hiPSCs (mix) at passage 20. Furthermore, no significant production of cytokines by any kind of hPSC or hPSC‐derived cardiomyocyte investigated in this study was observed when mononuclear cells were not added to hPSCs or hPSC‐derived cardiomyocytes (negative control experiments). These results indicate that the hiPSCs (mix) developed in this study show no immunogenic reactions and have transient universal characteristics during passage 10‐25 when transient universal hiPSCs (mix) contact allogenic mononuclear cells.

## 4 DISCUSSION

We hypothesize that any AF contains few ‘hypoimmunogenic or universal hAFSCs’ and many typical cells that are not hypoimmunogenic or universal hAFSCs and that hypoimmunogenic or universal hAFSCs do not express HLA class Ia and class II transiently after reprogramming of hypoimmunogenic or universal hAFSCs into hiPSCs as well as after the differentiation of transient universal hiPSCs. If this hypothesis is correct, the key point is how to select these ‘hypoimmunogenic or universal hAFSCs’ from a technical point of view. Our method in this study involved mixing AF in advance to generate hAFSCs (mix) by culturing the cells in mixed AF from different donors on conventional TCPS plates (Figure 1). Stem cells such as hAFSCs can be isolated from AF because of the high adhesion characteristics of stem cells on TCPS plates, while other cells cannot attach to the plates. When mixing AF from different pregnant women, mononuclear cells including CD8^+^ T cells, NK cells and macrophages or other components of AF from different individuals are thought to generate immunogenic reactions targeted to hAFSCs from different people, and only ‘hypoimmunogenic or universal hAFSCs’ survive after mixing AF from more than 2 donors. If the HLA class Ia type of the AF from 2 different individuals is matched, we expect that hypoimmunogenic or universal hiPSCs will not be generated. However, considering the probability of random matching of HLA class Ia, it was very unlikely that we would match the HLA class Ia types of any AF samples in our 8 experimental attempts (4 times × 2 different types of transient universal hiPSCs [hiPSCs (mix‐2) and hiPSCs (mix‐5)]) to generate transient universal hiPSCs from AF from multiple pregnant women. However, in the future, it might be safer to mix AF from more than 3 donors rather than mixing AF from only 2 donors for industrial production of transient universal hiPSCs via mismatching of HLA class Ia in AF obtained from different donors. This is because students in my laboratory generate hAFSCs by mixing AF derived from different pregnant women to speed up the preparation of hAFSCs compared to that of hAFSCs prepared from a single donor, and we succeeded in generating transient universal hiPSCs (mix) by reprogramming hAFSCs (mix) derived from a mixed source of AF when we checked the expression of HLA class Ia and class II on hiPSCs, although we expected hAFSCs (mix) might have permanent universal characteristics (hiPSCs (mix‐2) and hiPSCs (mix‐5) expressed no HLA class Ia even after 30 passages at first.

We hope that our transient universal hiPSCs at passage 10‐25 can be used for stem cell therapy in any patient using a single cell line without usage of immunosuppressive medicines in future. Since hiPSCs derived from a single AF donor do not show characteristics of hypoimmunogenic or universal hiPSCs, mixing AF derived from more than 2 donors with different HLA types is important to establish transient universal hiPSCs (mix). This phenomenon is considered to be originated from mononuclear cells containing in the allogenic AF that trigger apoptosis of allogenic AFSCs strongly expressing HLA class Ia after reprogramming into hiPSCs.

To verify our idea, conventional hESCs (H9, 10^5^ cells) were treated with allogenic mononuclear cells (10^5^ cells) for several times (1‐3 times) (Figure S7). HLA class Ia expression was gradually decreased with increase of mononuclear cell treatment where hESCs and mononuclear cells were incubated for 2 days in each mononuclear cell treatment (Figure S7B). hESCs after treated with mononuclear cells more than twice did not show HLA class Ia and class II (hypoimmunogenic or universal hESCs). Therefore, we verified that conventional hESCs contain hypoimmunogenic or universal hESCs, which do not show HLA class Ia and class II and we can isolate hypoimmunogenic or universal hESCs by allogenic mononuclear cell treatment in this study. However, it should be noted that hESCs treated with mononuclear cells start to express HLA class Ia after the passage without treatment of mononuclear cells. Therefore, this phenomenon is also found to be a transient issue.

It should be noted that our transient universal hiPSCs (mix) during specific passages (10‐25) and their differentiated cells into cardiomyocytes, MSCs and EBs that contain the cells derived all 3 germ layers did not show HLA class Ia and class II. Furthermore, the hiPSCs (mix) during specific passages (10‐25) and their differentiated cells did not show response to mononuclear cells where mononuclear cells contain 40% CD3^+^CD4^+^ T cells, 18% CD3^+^CD8^+^ T cells and 15% NK cells, and 15% monocytes including macrophages.^39^ Therefore, our transient universal hiPSCs (mix) and their differentiated cells are expected to show HLA‐E, HLA‐G, PD‐L1 and/or CD47 expression to escape NK cell and macrophage responses. This is probably because our original mother cells to generate hiPSCs are foetal cells (hAFSCs), which can escape immunogenic reaction from their mother tissues and cells. Our transient universal hiPSCs (mix) seem to hold genetic memory of hAFSCs until passage 25, because hAFSCs have immune privilege characteristics to mononuclear cells and express HLA‐G and PD‐L1.

We found that the selection of transient universal hAFSCs (mix), which are derived from multiple sources of AF and can be reprogrammed into hiPSCs (mix), is important to general our transient universal hiPSCs (mix) without genetic modification, such as clustered regularly interspaced short palindromic repeats (CRISPR)/CRISPR‐associated protein 9 (Cas9) gene‐editing technology.

Several researchers have considered developing universal or hypoimmunogenic hPSCs that do not express HLA Class Ia and Class II.^18^, ^19^, ^20^, ^21^, ^40^, ^41^, ^42^, ^43^, ^44^ However, all of these methods except the method presented in our present study require knocking out, knocking in or editing specific genes, which indicate the need for genetic modification (editing) of the cells, which restrict the clinical use of the hPSCs. For example, several researchers reported knocking out or gene editing HLA‐A, ‐B and/or ‐C genes to generate homozygous hPSCs.^18^, ^20^, ^23^, ^24^, ^25^ Xu et al knocked out HLA‐A and HLA‐B of hiPSCs, which originally have heterozygous HLA class I and they made hiPSCs having single HLA‐C alle using CRISPR/Cas9 gene‐editing technology.^20^

It is known that hPSCs expressing no HLA class I such as hPSCs knocked out β2‐microglobulin lead to the missing‐self response, which activate the lysing of the cells by NK cells.^18^, ^19^, ^20^, ^21^, ^23^ Several researchers knocked in HLA‐E, HLA‐G, PD‐L1 and/or CD47 genes to escape NK cell responses.^19^, ^21^ Han et al knocked out β‐2 microglobulin to prepare hESCs expressing no HLA type and knocked in PD‐L1 and HLA‐G on the AAVS1 safe harbour locus, which are immunomodulatory factors to escape NK cell response.^19^ They further knocked in CD47 genes, which is ‘donot‐eat me’ signal for macrophage on the AAVS1 safe harbour locus.

## 5 CONCLUSION AND FUTURE PERSPECTIVES

AF contains few ‘hypoimmunogenic or universal hAFSCs’, which can be reprogrammed into transient universal hiPSCs (mix). Mixing AF derived from different donors before the establishment of hAFSCs was the key selection technique to generate transient universal hiPSCs (mix) without genetic modification or gene editing in this study. The transient universal hiPSCs (mix) developed in this study were found to maintain their ‘universal’ characteristics (no expression of HLA Class Ia and Class II and no immunogenic reaction to cells originating from other individuals) during 10‐25 passages, even after differentiation into cardiomyocytes, MSCs or other lineages of 3 germ layer cells, as indicated by the characterization of mononuclear cell treatment of our transient universal hiPSCs (mix). Our transient universal hiPSCs (mix), which have designed and created without genetic modification in this study should be valuable after differentiation into photoreceptor cells, retinal pigment epithelial cells, TH^+^ cells, hepatocytes, haematopoietic stem cells, MSCs (haematopoietic stem cells in umbilical cord blood and MSCs started to be used for clinical trials to cure patients infected with COVID‐19, which enhance patient's immunity or treat lung functions for treatment of COVID‐19 or other unknown virus infection), β cells, and other tissue cells for future clinical usage of transient universal hiPSCs (mix).

## CONFLICT OF INTEREST

The authors declare no conflicts of interest.

## AUTHOR CONTRIBUTIONS

AH designed and organized the whole experimental plan and idea and wrote the manuscript. TCS and YPJ generated and characterized the universal hiPSCs. JYH isolated hAFSCs from amniotic fluid. QDL and SSK characterized the pluripotency of universal hPSCs. HC and QY discussed the characterization of universal hPSCs. YC prepared mononuclear cells from peripheral blood and performed mononuclear cell treatment of the cells. STH provided amniotic fluid and isolated hAFSCs from amniotic fluid.

## Supporting information

Supplementary Material

Video S1

Video S2

Video S3

Video S4

## Data Availability

The data that support the findings of this study are available from the corresponding author upon reasonable request.
