# Supplementary material for: Transient characteristics of universal cells on human‐induced pluripotent stem cells and their differentiated cells derived from foetal stem cells with mixed donor sources
Source: Cell Prolif. 2021 Feb 1;54(3):e12995. doi: 10.1111/cpr.12995 (PMC7941237; doi:10.1111/cpr.12995)
Supplement: Supplementary file 1 — Supplementary Material [file CPR-54-e12995-s002.docx]

**Transient characteristics of universal cells on human induced pluripotent stem cells and their differentiated cells derived from fetal stem cells with mixed donor sources**

Tzu-Cheng Sung^1,2†^, Yi-Peng Jiang^2†^, Jhe-Yu Hsu^2^, Qing-Dong Ling^3^, Hao Chen^1^, S. Suresh Kumar^4^, Yung Chang^5^, Shih-Tien Hsu^6^, Qingsong Ye^7-9,*^, Akon Higuchi^1,2,5,10,11*^

^1^School of Ophthalmology and Optometry, Eye Hospital, Wenzhou Medical University, Wenzhou, Zhejiang, 325027, China

^2^Department of Chemical and Materials Engineering, National Central University, Taoyuan, Taiwan, R.O.C.

^3^Cathay Medical Research Institute, Cathay General Hospital, Taipei, Taiwan, R.O.C.

^4^Department of Medical Microbiology and Parasitology, Universiti Putra Malaysia, Slangor, Malaysia

^5^Department of Chemical Engineering and R&D Center for Membrane Technology, Chung Yuan Christian University, Taoyuan, Taiwan, R.O.C.

^6^Department of Internal Medicine, Taiwan Landseed Hospital, Pingjen City, Taiwan, R.O.C.

^7^Center of Regenerative Medicine, Renmin Hospital of Wuhan University, Hubei, China

^8^School and Hospital of Stomatology, Wenzhou Medical University, Wenzhou, Zhejiang, China

^9^Skeletal Biology Research Center, Department of Oral Maxillofacial Surgery, Massachusetts General Hospital & Harvard School of Dental Medicine, Boston, MA, USA

^10^Wenzhou Institute, University of Chinese Academy of Science, Wenzhou, China

^11^Nano Medical Engineering Laboratory, Riken Cluster for Pioneering Research, Riken, Wako, Japan

^†^ These authors contributed equally.

| **Table S1** Materials used in this study. | | | |
| --- | --- | --- | --- |
| Materials | Abbreviation | Catalog No. | Company |
| **ECM** | | | |
| Matrigel | Matrigel | #356230 | Corning (Corning, NY, USA) |
| Recombinant vitronectin | rVN | A14700 | Thermo Fisher Scientific Inc. (Waltham, MA, USA) |
|  |  |  |  |
| **Cell culture dishes** | | | |
| 6-well tissue culture polystyrene plate | TCPS | #353046 | Corning (Corning, NY, USA) |
| 3.5 cm tissue culture polystyrene dishes | TCPS | #353001 | Corning (Corning, NY, USA) |
| Ultra-low attachment plate | Ultra-low attachment plate | #3471 | Corning (Corning, NY, USA) |
|  |  |  |  |
| **Chemicals** | | | |
| Dispase II | Dispase | D4693-1G | Sigma-Aldrich (St. Louis, MO, USA) |
| Ficoll-Paque |  |  |  |
| Hochest 33342 | Hochest | PA-3014 | Lonza (Basel, Switzerland) |
| 7-AAD |  |  |  |
|  |  |  |  |
| **Cell culture medium and component** | | | |
| Essential 8 medium | Essential 8 | A1517001 | Thermo Fisher Scientific Inc. (Waltham, MA, USA) |
| Essential 6 medium | Essential 6 | A1516401 | Thermo Fisher Scientific Inc. (Waltham, MA, USA) |
| MCDB 201 medium | MCDB 201 medium | M6770 | Sigma-Aldrich (St. Louis, MO, USA) |
| DMEM medium | DMEM medium | D5648-10x1L | Sigma-Aldrich (St. Louis, MO, USA) |
| DMEM/F12 medium | DMEM/F12 medium | 11330-057 | Thermo Fisher Scientific Inc. (Waltham, MA, USA) |
| RPMI 1640 | RPMI 1640 | 11875093 | Thermo Fisher Scientific Inc. (Waltham, MA, USA) |
| FBS | FBS | 04-001-1A | Biological Industries (Kibbutz Beit-Haemek, Israel) |
| FGF-2 |  |  |  |
| B-27™ Supplement, minus insulin | B27- | A1895601 | Thermo Fisher Scientific Inc. (Waltham, MA, USA) |
| B-27™ Supplement | B27 | 17504044 | Thermo Fisher Scientific Inc. (Waltham, MA, USA) |
| CHIR99021 | CHIR99021 | SML1046 | Sigma-Aldrich (St. Louis, MO, USA) |
| IWR-1 | IWR-1 | I0161 | Sigma-Aldrich (St. Louis, MO, USA) |
| Live/Dead cell imaging kit | Live/Dead cell imaging kit | R37601 | Thermo Fisher Scientific Inc. (Waltham, MA, USA) |
| CytoTune^R^-iPS 2.0 Sendai Reprogramming Kit | CytoTune^R^-iPS 2.0 | [A16518](https://www.thermofisher.com/order/catalog/product/A16518) | Thermo Fisher Scientific Inc. (Waltham, MA, USA) |

| **Table S1** (continued) Materials used in this study. | | | |
| --- | --- | --- | --- |
| Materials | Abbreviation | Catalog No. | Company |
| **Antibodies** | | | |
| Anti-Oct3/4 antibody | Anti-Oct3/4 antibody | sc-5279 | Santa Cruz Biotechnology (Dallas, TX, USA) |
| Anti-Sox2 antibody | Anti-Sox2 antibody | AB5603 | Merck KGaA (Darmstadt, Germany) |
| Anti-SSEA-4 antibody | Anti-SSEA-4 antibody | ab16287 | Abcam (Cambridge, MA, USA) |
| Anti-Nanog antibody | Anti-Nanog antibody | MA1-017 | Thermo Fisher Scientific Inc. (Waltham, MA, USA) |
| Anti-AFP antibody | Anti-AFP antibody | PA5-21004 | Thermo Fisher Scientific Inc. (Waltham, MA, USA) |
| Anti-SMA antibody | Anti-SMA antibody | PA5-19465 | Thermo Fisher Scientific Inc. (Waltham, MA, USA) |
| Anti-GFAP antibody | Anti-GFAP antibody | MA5-15086 | Thermo Fisher Scientific Inc. (Waltham, MA, USA) |
| Alexa Fluor 488 goat anti-mouse IgG | Alexa Fluor 488 goat anti-mouse IgG | A11001 | Thermo Fisher Scientific Inc. (Waltham, MA, USA) |
| Alexa Fluor 488 goat anti-rabbit IgG | Alexa Fluor 488 goat anti-rabbit IgG | A11008 | Thermo Fisher Scientific Inc. (Waltham, MA, USA) |
| Alexa Fluor 594 donkey anti-rabbit IgG | Alexa Fluor 594 donkey anti-rabbit IgG | A21207 | Thermo Fisher Scientific Inc. (Waltham, MA, USA) |
| Anti-MLC2a antibody | Anti-MLC2a antibody | ab92721 | Abcam (Milton, Cambridge, UK) |
| Anti-cTnT antibody | Anti-cTnT antibody | MA5-12960 | Thermo Fisher Scientific Inc. (Waltham, MA, USA) |
| Anti-HLA Class 1 ABC antibody [W6/32], prediluted (PE/Cy5®) | Anti-HLA Class I antibody | ab155381 | Abcam (Cambridge, MA, USA) |
| Anti-HLA DR + DP + DQ antibody [WR18] (Phycoerythrin) | Anti-HLA Class II antibody | ab23901 | Abcam (Cambridge, MA, USA) |
| Mouse IgG2a kappa isotype control, (PE/Cy5®) | Anti-HLA Class I isotype antibody | 15-4724-81 | Thermo Fisher Scientific Inc. (Waltham, MA, USA) |
| Mouse IgG2a [X5563] (Phycoerythrin) - Isotype Control | Anti-HLA Class II isotype antibody | ab91363 | Abcam (Cambridge, MA, USA) |
|  |  |  |  |
|  | **Mice** |  |  |
| NOD.CB17-Prkdcscid/Jnarl | NOD-SCID mice | NOD.CB17-Prkdcscid/Jnarl | National Laboratory Animal Center (Taipei, Taiwan) |


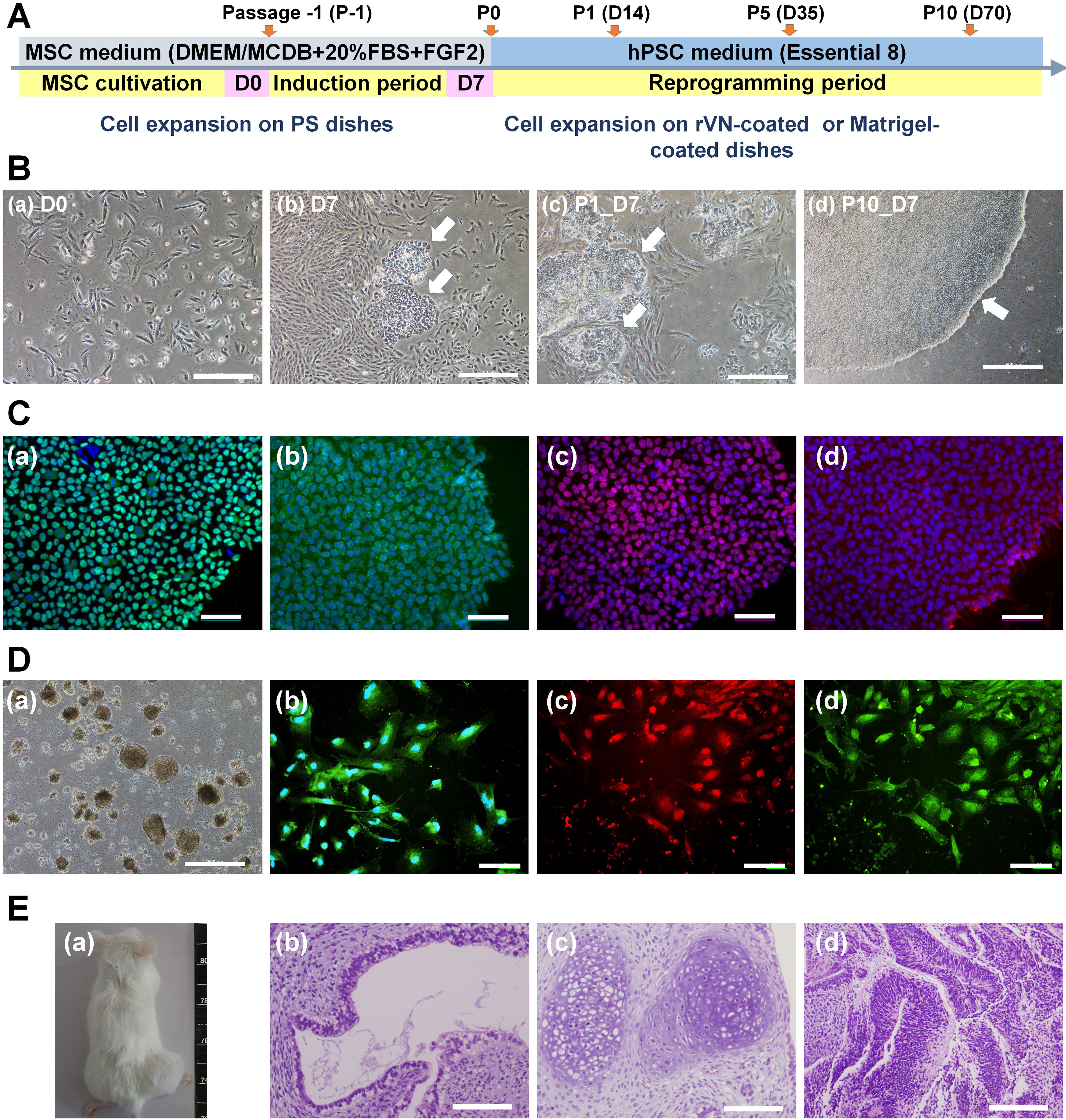


FIGURE S1 Generation of transient universal hiPSCs (mix-2). (A) Preparation timeline of transient universal hiPSCs (mix-2) using hAFSCs. (B) The sequential morphological changes during reprogramming of hAFSCs into transient universal hiPSCs (mix-2) at day 0 of passage 0 (a), day 7 of passage 0 (b), day 7 of passage 1 (c), and day 7 of passage 10 (d). The scale bar indicates 500 μm. (C) Expression of the pluripotency proteins Oct4 (a, green), Sox2 (b, green), Nanog (c, red), and SSEA-4 (d, red) in transient universal hiPSCs (mix-2) evaluated by immunostaining, with nuclear staining with Hoechst 33342 (blue) after culturing for 20 passages. The scale bar indicates 50 μm. (D) (a) Morphology of cells from EBs differentiated from hiPSCs (mix-2) after culturing for 21 passages. Expression of a mesodermal protein (b, SMA, green), an ectodermal protein (c, GFAP, red), and an endodermal protein (d, AFP, green) in the cells shown by immunostaining with nuclear staining with Hoechst 33342 (b, blue), after culturing for 21 passages. The scale bar indicates 500 μm (a) and 100 μm (b-d). (E) A teratoma was formed by injecting transient universal hiPSCs (mix-2) cultured on recombinant vitronectin-coated dishes after 22 passages (a). Tissues including the gland duct consisting of the columnar epithelium (b, endoderm), cartilage (c, mesoderm), and immature neuroepithelium (d, ectoderm) can be observed. The scale bar indicates 200 μm (b-d).


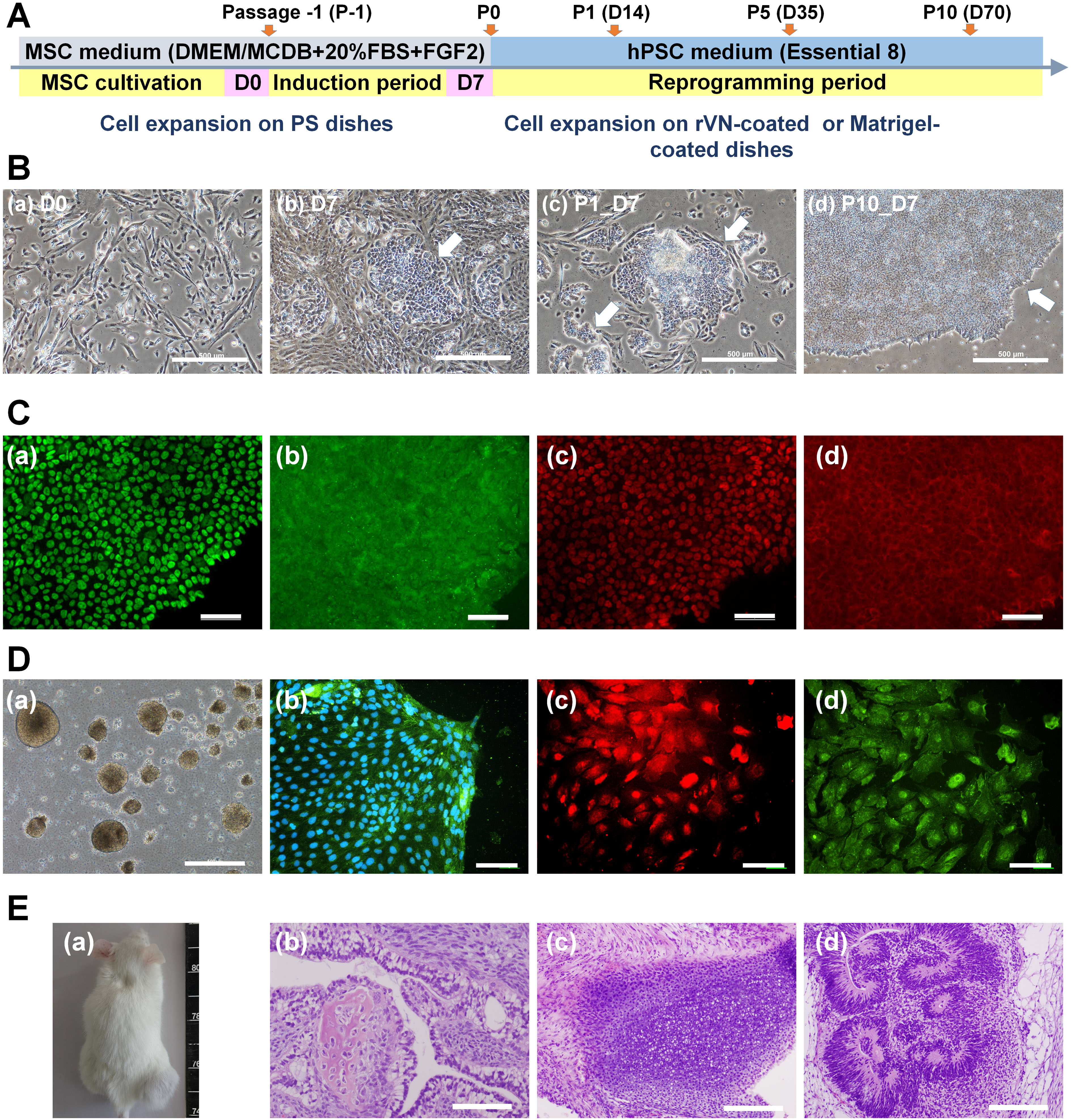


FIGURE S2 Generation of hiPSCs (single) using single donor of AF. (A) Preparation timeline of hiPSCs (single) using hAFSCs. (B) The sequential morphological changes during reprogramming of hAFSCs into hiPSCs (single) at day 0 of passage 0 (a), day 7 of passage 0 (b), day 7 of passage 1 (c), and day 7 of passage 10 (d). The scale bar indicates 500 μm. (C) Expression of the pluripotency proteins Oct4 (a, green), Sox2 (b, green), Nanog (c, red), and SSEA-4 (d, red) in hiPSC (single) evaluated by immunostaining, with nuclear staining with Hoechst 33342 (blue) after culturing for 20 passages. The scale bar indicates 50 μm. (D) (a) Morphology of cells from EBs differentiated from hiPSC(single) after culturing for 21 passages. Expression of a mesodermal protein (b, SMA, green), an ectodermal protein (c, GFAP, red), and an endodermal protein (d, AFP, green) in the cells shown by immunostaining with nuclear staining with Hoechst 33342 (b, blue) after culturing for 21 passages. The scale bar indicates 500 μm (a) and 100 μm (b-d). (E) A teratoma was formed by injecting hiPSC (single) cultured on recombinant vitronectin-coated dishes after 22 passages (a). Tissues including the gland duct consisting of the columnar epithelium (b, endoderm), cartilage (c, mesoderm), and immature neuroepithelium (d, ectoderm) can be observed. The scale bar indicates 200 μm (b-d).


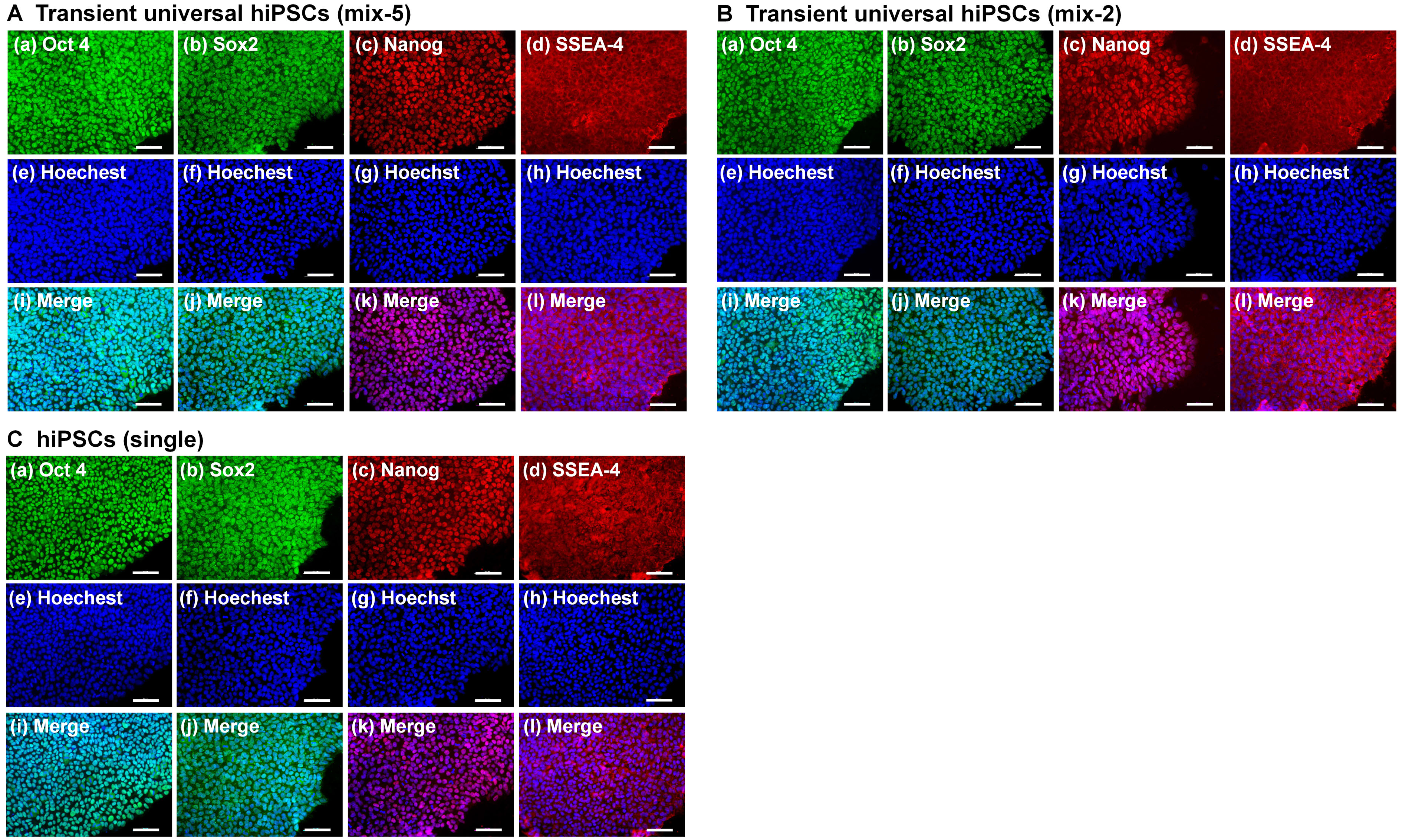


FIGURE S3 Characterization of the pluripotency of transient universal hiPSCs and hiPSCs(single). Pluripotency of transient universal hiPSCs (mix-5) (A), transient universal hiPSCs (mix-2) (B) and hiPSCs (single) (C), which were cultured on recombinant vitronectin-coated dishes for 21 passages. Expression of the pluripotency proteins Oct4 (a, green), Sox2 (b, green), Nanog (c, red), and SSEA-4 (d, red) in hiPSCs evaluated by immunostaining, with nuclear staining with Hoechst 33342 (blue, e-h) after culturing for 21 passages. The photos (i), (j), (k), and (l) were generated by merging (a) and (e), (b) and (f), (c) and (g), and (d) and (h), respectively. The scale bar indicates 50 μm.


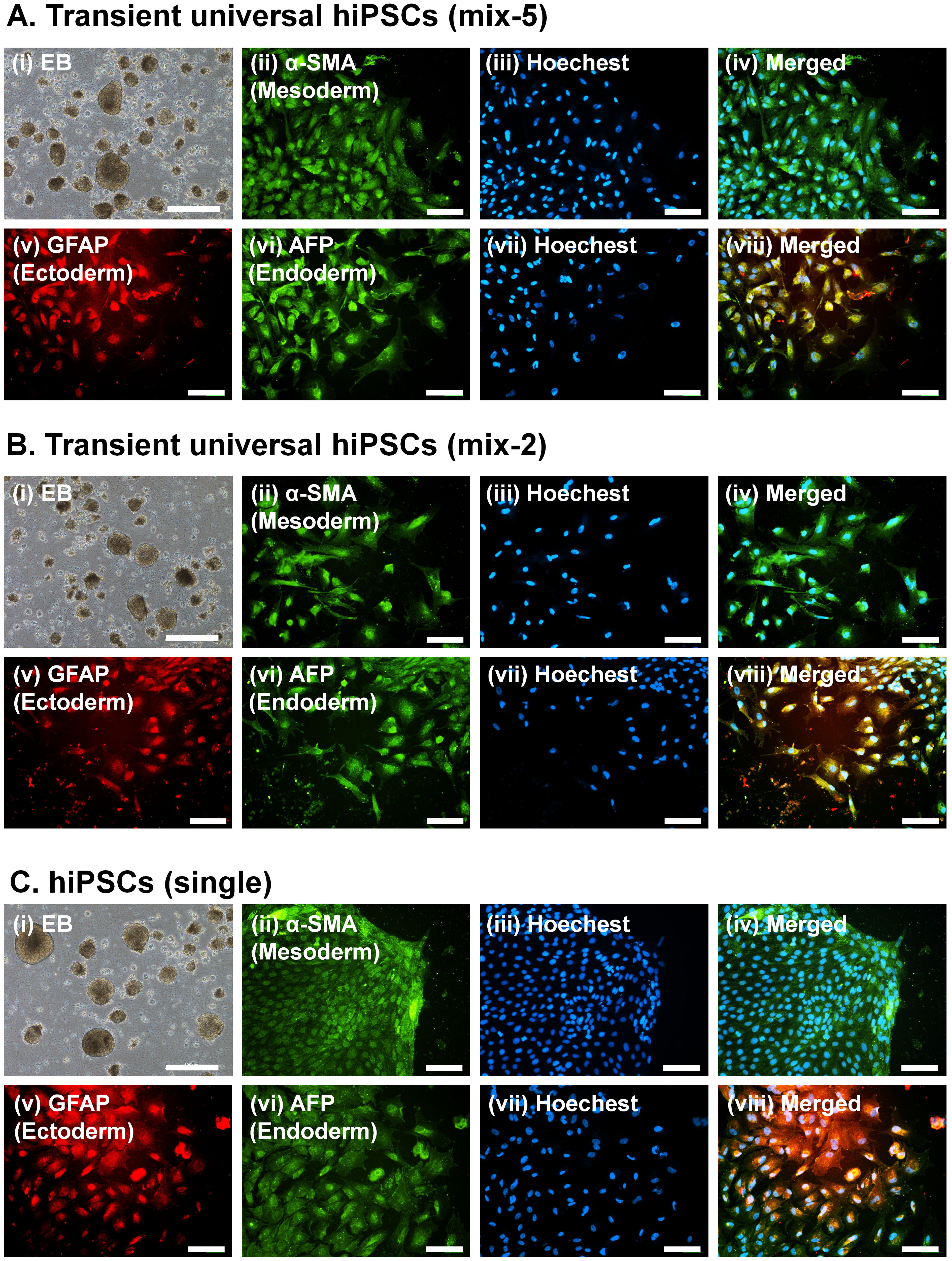


FIGURE S4 Characterization of the differentiation capability in vitro of transient universal hiPSCs (mix-5) (A), transient universal hiPSCs (mix-2) (B), and hiPSCs derived from single donor of AF (hiPSC (single)) (C). (i) Morphology of cells from EBs differentiated from hiPSCs after culturing for 20 passages. Expression of a mesodermal protein (ii, SMA, green), an ectodermal protein (v, GFAP, red), and an endodermal protein (vi, AFP, green) in hiPSCs shown by immunostaining with nuclear staining with Hoechst 33342 (iii and vii, blue) after culturing for 20 passages. The photo (iv) was generated by merging (ii) and (iii). The photo (vii) was generated by merging (v), (vi), and (vii). The scale bar indicates 500 μm (i) and 100 μm (ii-viii).


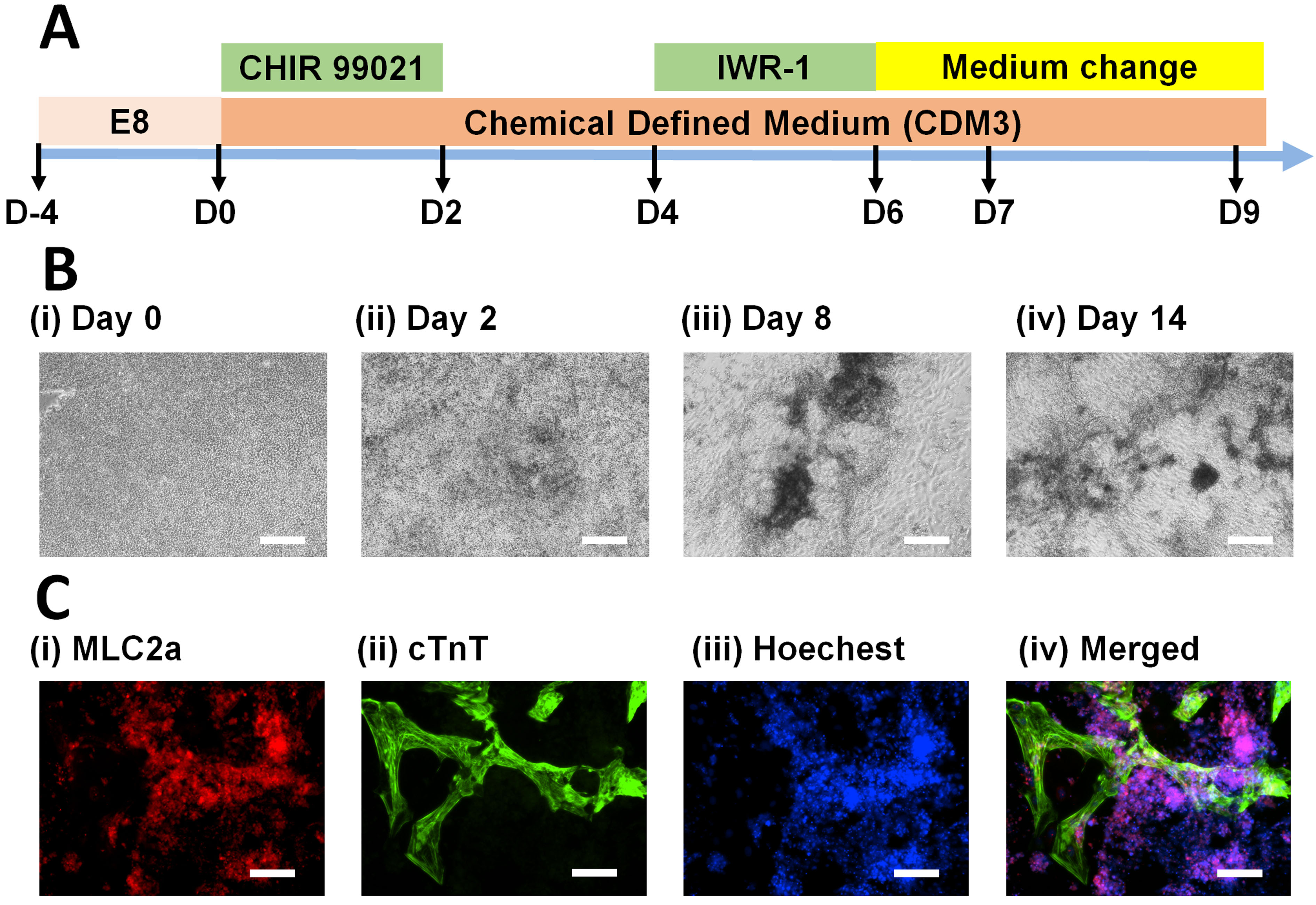


FIGURE S5 Cardiomyocytes differentiation of hiPSCs (single) at passage 20. (A) Timelines of the protocols for hiPSC differentiation into cardiomyocytes. (B) The sequential morphological changes during hiPSCs (single) differentiation towards the cardiac lineage at day 0 (i) day 2 (ii), day 8 (iii) and day 14 (iv). The bar indicates 100 μm. (C) Expression of MLC2a (i, red) and cTnT (ii, green) in hiPSCs (single)-derived cardiomyocytes analyzed by an immunostaining method after 14 days of induction. The nuclei were stained with Hoechst 33342 (iii, blue). The photo (iv) was generated by merging (i)–(iii). The bar indicates 100 μm.


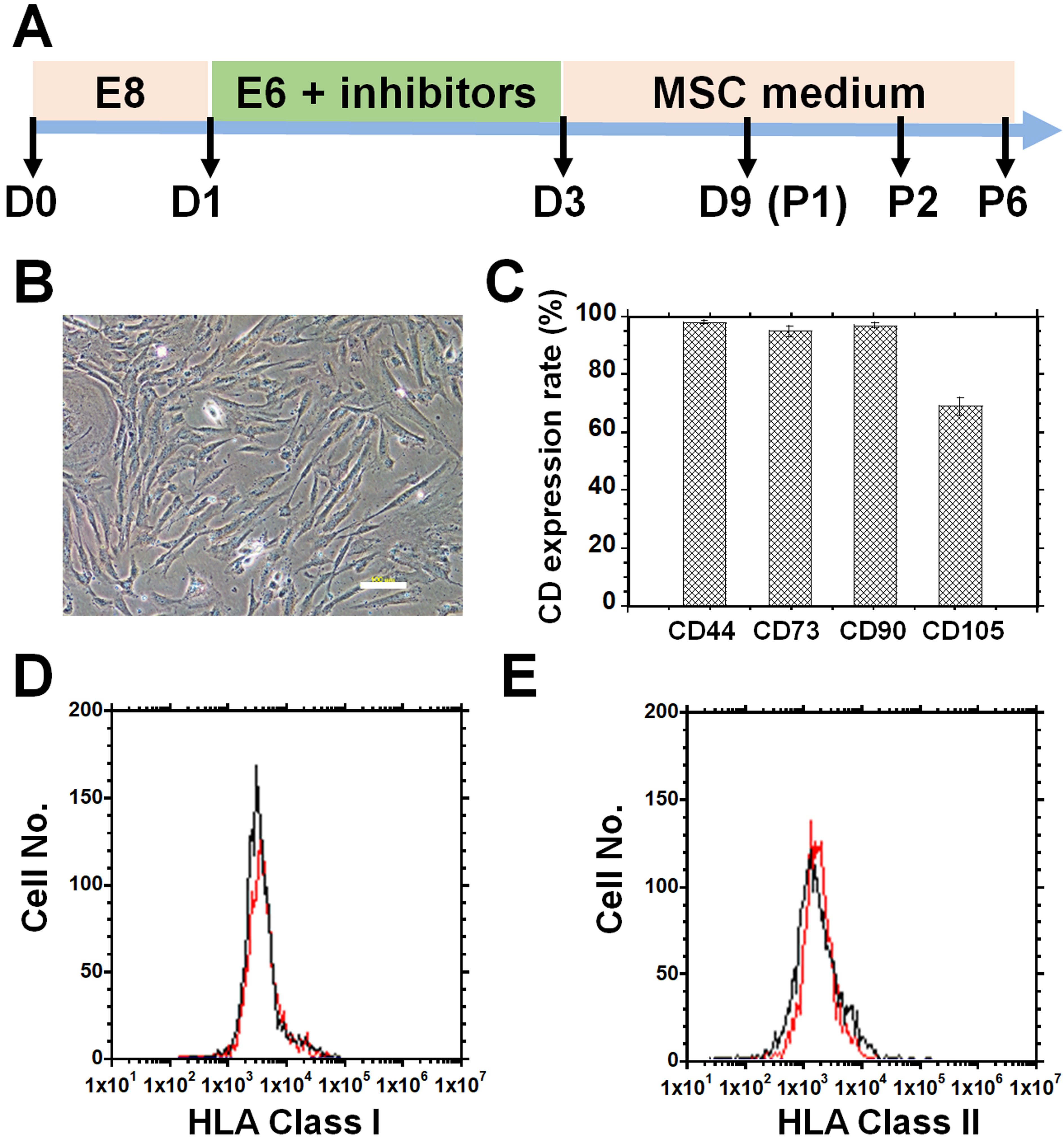


FIGURE S6 MSCs derived from transient universal hiPSCs at passage 25 shows no HLA Class Ia and Class II. (A) Timelines of the protocols for transient universal hiPSC (mix-5) differentiation into MSCs. (B) Morphology of transient universal hiPSC (mix-5)-derived MSCs. The scale bar indicates 100 μm. (C) MSC surface marker (CD44, CD73, CD90, and CD105) of transient universal hiPSC (mix-5)-derived MSCs. (D) HLA Class I expression of transient universal hiPSC (mix-5)-derived MSCs. (E) HLA Class II expression of transient universal hiPSC (mix-5)-derived MSCs.


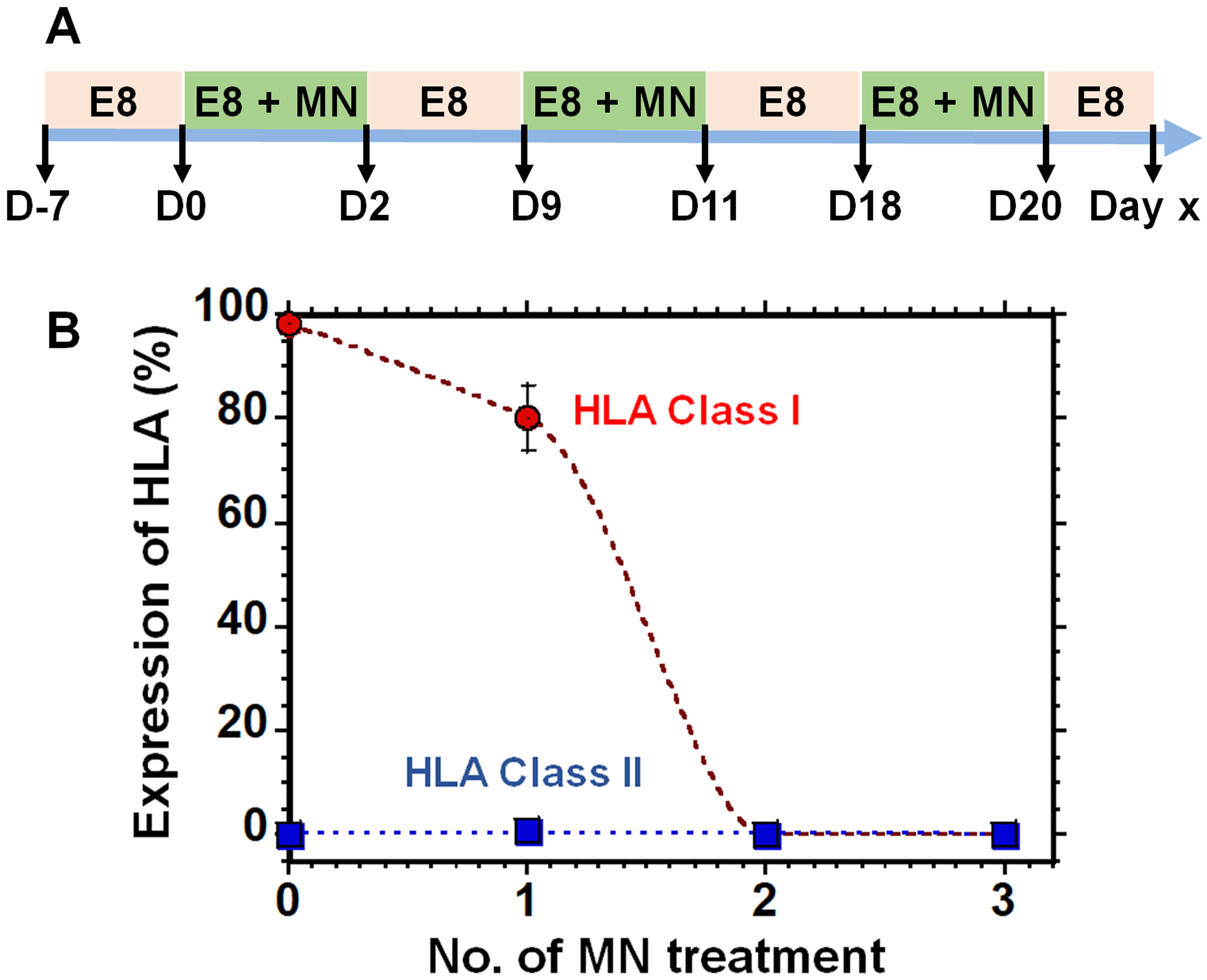


**FIGURE S7** Generation of transient universal hESCs. (A) Timeline of preparation method of transient universal hESCs. hESCs (H9) were treated with mononuclear cells triple time to delete hESCs expressing HLA Class Ia. (B) Dependence of HLA Class Ia and Class II of hESCs on the number of mononuclear cell (MN) treatment for generation of transient universal hESCs.

Movie Legend

Movie S1 Cardiomyocytes derived from transient universal hiPSCs (mix-2)

Movie S2 Cardiomyocytes derived from transient universal hiPSCs (mix-5)

Movie S3 Cardiomyocytes derived from transient universal hiPSCs (mix-2) after treatment with mononuclear cells

Movie S4 Cardiomyocytes derived from transient universal hiPSCs (mix-5) after treatment with mononuclear cells
